# Robust Bayesian hierarchical models for basket trials enabling joint evaluation of toxicity and efficacy

**DOI:** 10.1177/09622802261465336

**Published:** 2026-09-02

**Authors:** Zhi Cao, Pavel Mozgunov, Haiyan Zheng

**Affiliations:** 1 MRC Biostatistics Unit, 47959 University of Cambridge, Cambridge, UK; 2 Department of Mathematical Sciences, 1555 University of Bath - Claverton Down Campus, Bath, UK

**Keywords:** Bivariate analysis, borrowing strength, exchangeability, phase II, robustness

## Abstract

Basket trials have gained increasing attention for their efficiency, as multiple patient subgroups are evaluated simultaneously. Conducted basket trials focus primarily on establishing the early efficacy of a treatment, yet continued monitoring of toxicity is essential. In this article, we propose two Bayesian hierarchical models that enable bivariate analyses of toxicity and efficacy, while accounting for heterogeneity present in the treatment effects across patient subgroups. Specifically, one assumes the subgroup-specific toxicity and efficacy treatment effects, as a parameter vector, can be exchangeable or non-exchangeable; the other allows either the toxicity or efficacy parameters specific to the subgroups, to be exchangeable or non-exchangeable. The bivariate exchangeability and non-exchangeability distributions introduce a correlation parameter between treatment effects, while we stipulate a zero correlation when only toxicity or efficacy parameters are exchangeable. Simulation results show that our models perform robustly under different scenarios compared to the standard Bayesian hierarchical model and the stand-alone analyses, especially in producing higher power when the subgroup-specific effects are exchangeable in toxicity or efficacy only. When considerable correlation between the toxicity and efficacy effects exists, our methodology gives small error rates and greater power than alternatives that analyse toxicity and efficacy by parts.

## 1. Introduction

Precision medicine has been recognised as a new frontier for healthcare due to the rapid development of genetics and biomedical techniques.^1,2^ Patient heterogeneity is a key consideration for tailoring treatments based on the disease features. Consequently, the paradigm of drug development gradually moves away from one-size-fits-all approaches.^3^ In recent decades, innovative trial methodologies such as *master protocols* have emerged as feasible and efficient approaches to drug development in the era of precision medicine. Specifically, master protocols can streamline the design and analysis of clinical trials involving multiple patient strata to receive one or more treatments in a single overarching study.^4^

Basket trials are one major type of master protocols in clinical trials: a new treatment can be evaluated simultaneously in several patient subgroups sharing a common disease feature which can, for example, be the same genetic aberration.^5^ For such commonality, borrowing of information across patient subgroups is a preferred approach to the analysis of basket trials. Thall et al.^6^ discussed a Bayesian hierarchical model to estimate the efficacy parameters specific to subgroups under the assumption of exchangeability. Explicitly, subgroup-specific treatment effects are assumed as random samples from the same underlying distribution and the joint distribution remains invariant irrespective to their permutations.^7^ Such hierarchical structure is commonly implemented for information borrowing. However, assuming the exchangeability of all treatment effects can be overly restrictive, which can lead to inaccurate estimates for subtrials with extreme observations. Here, subtrials are formed by the patient subgroups each within a basket trial. To alleviate the concern, Neuenschwander et al.^8^ proposed a robust extension that accounts for possibility that each of the subtrial-specific parameter may be non-exchangeable with the others. Other innovative approaches for robust borrowing of information have been under development these years. For example, Hobbs and Landin^9^ proposed exchangeability monitoring strategy using Bayesian monitoring rules for sharing information. Zheng and Wason^10^ recommended information borrowing based on pair-wise discrepancies of treatment effects (monitoring the similarity of each pair of two subtrials to borrow information across those with high similarity). Besides, the adaptive clustering methods presuming more than one underlying distributions^11^ have been proposed to facilitate drug development.

Basket trials are often conducted in phase II oncology settings to evaluate the efficacy of an anti-cancer therapy, which targets specific molecular alterations across different tumour types. Oncologic basket trials are predominantly non-randomised, with all patients within a subtrial being treated with the single evaluated treatment (the so-called single-arm design) for rapid assessment of efficacy. Notable examples include the BRAF-V600 cancer study utilising vemurafenib, which demonstrated its applicability as a targetable oncogene across multiple malignancies.^12^ Recently, there is growing recognition to expand the scope of traditional basket trials to later phases and randomisations.^5,13^ The pursuit of higher levels of evidence for a broader clinical adoption also necessitates the use of randomised controlled designs. The MAJIC study (ISRCTN61925716) is a phase II randomised controlled basket trial designed to assess the long-term comparative safety and efficacy of ruxolitinib versus best available therapy in treating two types of blood cancers.^14^

The application of basket trials has extended beyond oncology to therapeutic areas such as neurodegenerative diseases. For instance, in neurodegenerative disorders research, basket trials have the potential to optimise resource use by testing various interventions within a unified framework.^15^ Recent advancements have also introduced the methodology exploration of basket trials to the confirmatory setting, such as early frequentist contributions including Chen et al.^16^ and He et al.^17^ to control type I error or family wise error rate (FWER). In the exploratory setting, IMPACT II (NCT02152254) randomises patients to a molecularly guided targeted therapy strategy versus standard-of-care, providing a comparative estimate of benefit and safety. A further motivating example in chronic diseases is the OACS trial (ISRCTN15223158, ISRCTN98936300), a randomised basket study of obeticholic acid versus placebo for cognitive dysfunction across subtrials in primary biliary cholangitis and Parkinson’s disease, using a continuous cognitive composite endpoint.

In drug development, phase II clinical trials answer the question of whether the new treatment can be recommended to the confirmatory phase III trial. There is growing recognition that solely evaluating efficacy in phase II trials may neglect other crucial aspects, such as safety, tolerability, and broader pharmacodynamic responses.^18^ This can lead to misjudgments about a drug’s overall viability, especially when safety and other effects remain inadequately explored. Therefore, efficacy outcomes in Phase II trials may not always translate to phase III success due to these overlooked factors. As an example, Chi et al.^18^ proposed a generalised calibrated Bayesian hierarchical model for a single-arm basket trial with joint evaluation of patient toxicity and efficacy of drug combinations (NCT05082025) which demonstrates a motivation to develop potential modelling strategies. Besides, current statistical methodology to formally monitor toxicity data generally concentrates on single experimental treatment arm. Ivanova et al.^19^ proposed a method to continuously monitor toxicity in phase II trials as early-stopping rules. Ray and Rai^20^ developed a mechanism combining toxicity monitoring with Simon’s two-stage design. More recently, a Bayesian multinomial model^21^ was established to jointly analyse discrete toxicity and efficacy. However, there is limited investigation concerning a formal appraisal of toxicity effects in phase II basket trials.

In this article, we develop robust bivariate Bayesian analysis models for phase II basket trials to jointly evaluate toxicity and efficacy outcomes. The toxicity and efficacy effects will be formulated to allow a robust information borrowing across patient subgroups. Our proposed methods can be reviewed as the extensions of the exchangeable–non-exchangeable (EXNEX) model by Neuenschwander et al.^8^: flexible prior mixture weights can be assigned to the exchangeability or non-exchangeability distributions of toxicity and efficacy parameters that imply different borrowing structures. Possible correlations between the toxicity and efficacy parameters are also taken into consideration in the bivariate analysis.

The remainder of this article is structured as follows. In Section 2, we describe how to model the basket trial data for a joint evaluation of toxicity and efficacy treatment effects. Two Bayesian models are proposed with consideration of robust information borrowing and inference. Next we present a simulation study to assess the operating characteristics in comparisons with alternative methods. Finally, Section 4 will present a discussion on the proposed approaches.

## 2. Methods

Consider a phase II randomised controlled basket trial with K subtrials, wherein patients are randomised to receive either the new treatment or control. Each subtrial is characterised by a patient subgroup of specific disease subtype. Figure 1 visualises the configuration of such randomised controlled basket trials.

**Figure 1.**
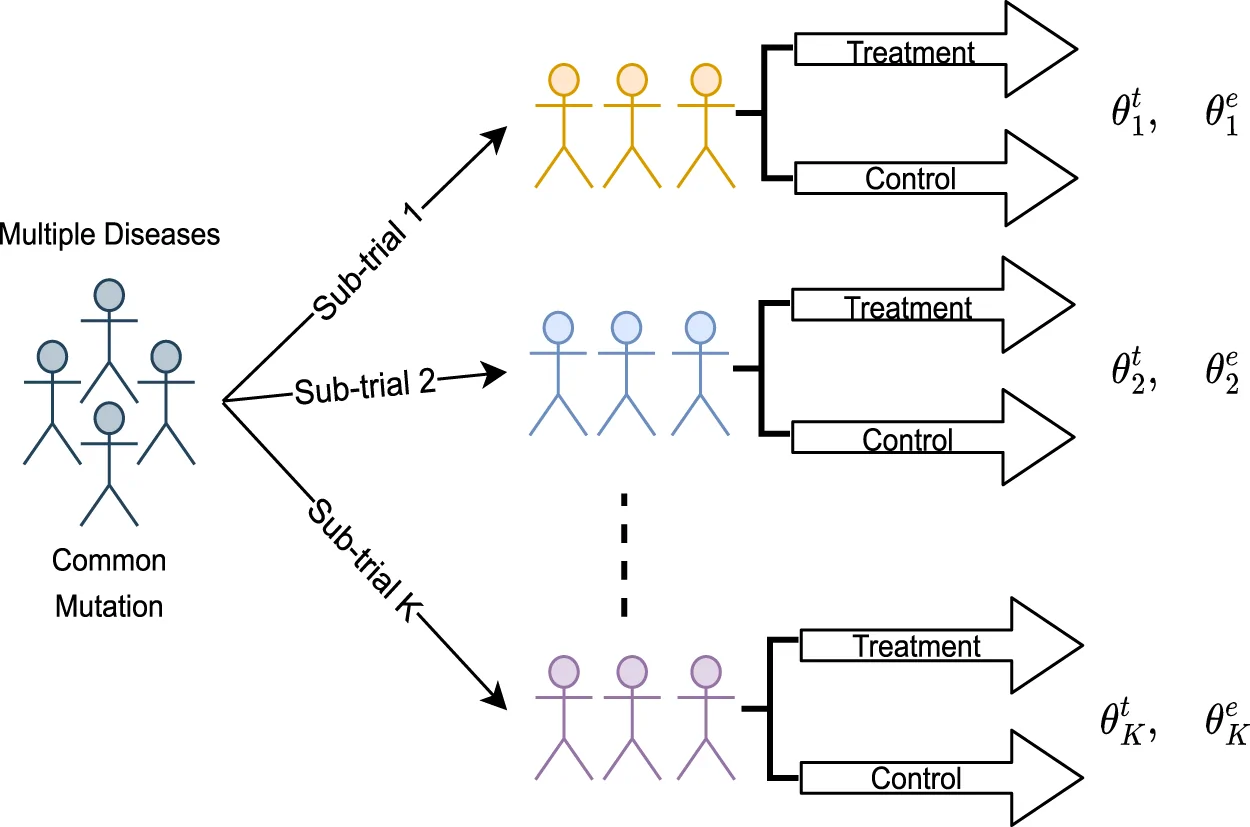
Randomised controlled basket trial. Patients are divided into *K* subtrials, denoted by different colours. Notations: $\theta_{k}^{t}$ and $\theta_{k}^{e}$ used to denote the toxicity and efficacy-related effects specific to subtrial k=1,2,…,K.

Let $n_{jk}$ be the sample size for subtrial k=1,2,…,K , where j=E,C labels the experimental or control group. We assume a binary toxicity outcome which aligns with the typical use of dose limiting toxicities (DLT), say, 0 for no DLT and 1 for a DLT, based on Common Terminology Criteria for Adverse Events (CTCAE).^22^ As an example, the DLT could be defined as any treatment-related adverse event (AE) of grade 3 or higher (severe or not immediately life-threatening). Beyond oncology, the idea to define binary toxicity is similarly based on dose limiting event (DLE). The efficacy endpoint, in particular, is assumed to be continuous since in the era of precision medicine there is more recognition of using continuous efficacy biomarkers including tumor size shrinkage,^23^ and ctDNA (referring to the small fragments of DNA released by tumor cells into the bloodstream) which is increasingly used to monitor tumour’s growth,^24^ and so forth.

### 2.1. Data models for toxicity and efficacy outcomes

Let $y_{jk}$ be the number of toxicity responses in treatment group j within subtrial k , which follows a $\text{Binomial}(n_{jk},p_{jk})$ distribution with the response probability of $p_{jk}$ . We employ the binomial model with the ‘logit’ link function: for k=1,…,K ,

(1) $$\begin{array}{rl} & y_{jk}∼\text{Binomial}\left(n_{jk},p_{jk}\right)\\ & \text{logit}(p_{jk})=\alpha_{k}^{t}+T(j)\cdot \theta_{k}^{t} \end{array}$$

where T(j) is the indicator function which equals 1 if j=E or 0 if j=C . After the logit transformation, we can model the unknown baseline effect $\alpha_{k}^{t}$ (superscript t for toxicity indication), and the toxicity treatment effect $\theta_{k}^{t}$ .

Assuming normally distributed data, let $z_{ijk}$ be the efficacy outcome for patient i receiving treatment j , within subtrial k . A patient-level efficacy model can be established as

(2) $$\begin{array}{rl} & z_{ijk}∼N(\mu_{jk},\sigma_{jk}^{2})\\ & \mu_{jk}=\alpha_{k}^{e}+T(j)\cdot \theta_{k}^{e} \end{array}$$

Unlike the toxicity model, the identity link is chosen, giving rise to the equivalence between the baseline effect $\alpha_{k}^{e}$ (superscript e for efficacy indication) and the mean responses for the control group $\mu_{Ck}$ in subtrial k . Efficacy treatment effect $\theta_{k}^{e}$ can be established similarly to the toxicity model.

### 2.2. Bivariate EXNEX (BiEXNEX) model

Building upon the idea of EXNEX model by Neuenschwander et al.,^8^ we propose a bivariate Bayesian hierarchical model allowing for robust information borrowing across patient subgroups to jointly analyse the toxicity and efficacy effects. We also consider possible correlations between the two effects in the modelling.

The first hierarchical model (bivariate EXNEX, abbreviated to BiEXNEX hereafter) is established by assuming the subtrial-specific parameter vectors of toxicity and efficacy effects are either exchangeable (EX) or non-exchangeable (NEX). That is, for each subtrial k : with prior probability $\omega_{k}$ , parameter vectors are EX as follows:

(3a) $$\left(\begin{array}{c} \theta_{k}^{t}\\ \theta_{k}^{e} \end{array}\right)|\left(\begin{array}{c} \beta_{1}\\ \beta_{2} \end{array}\right),\;\left(\begin{array}{c} \phi_{1}\\ \phi_{2} \end{array}\right),\;\rho ∼N\left(\left(\begin{array}{c} \beta_{1}\\ \beta_{2} \end{array}\right),\left(\begin{array}{cc} \phi_{1}^{2} & \rho \phi_{1}\phi_{2}\\ \rho \phi_{1}\phi_{2} & \phi_{2}^{2} \end{array}\right)\right)$$

with prior probability $1-\omega_{k}$ , parameter vectors are NEX as follows:

(3b) $$\left(\begin{array}{c} \theta_{k}^{t}\\ \theta_{k}^{e} \end{array}\right)|\kappa \;∼\;N\left(\left(\begin{array}{c} m_{1k}\\ m_{2k} \end{array}\right),\left(\begin{array}{cc} s_{1k}^{2} & \kappa s_{1k}s_{2k}\\ \kappa s_{1k}s_{2k} & s_{2k}^{2} \end{array}\right)\right)$$

This BiEXNEX model contains prior probability of exchangeability, $\omega_{k}\in (0,1)$ , for each subtrial k , which may be specified according to historical knowledge or advice from practitioners. The unknown parameters $(\beta_{1},\beta_{2}{)}^{T}$ and $(\phi_{1},\phi_{2}{)}^{T}$ are the mean vector and standard deviations in the covariance matrix of the EX part shared by all subtrials, with ρ denoting the unknown correlation following the uniform prior U(−1,1) . $\beta_{1}$ and $\beta_{2}$ can be inferred with a normally distributed prior $N(0,b^{2})$ , where b is the prior standard deviation. The general prior selection for β can be uninformative or weakly informative according to prior knowledge and clinicians’ perspectives. For uninformative priors, b can be set to a very large value, ensuring the toxicity difference between control and treatment groups covers a broad range of values. The prior standard deviations for toxicity and efficacy can be set differently in practice to reflect differing expected heterogeneity of the two endpoints. Specifying priors for $\phi_{1}^{2}$ and $\phi_{2}^{2}$ is critical in the Bayesian setting – it can be interpreted as the heterogeneity between patient subgroups. Half-normal, half-Cauchy and half-t distributions can be chosen as candidates.^25–27^ We assume it follows a half-normal distribution Half-N(x) , where x determines the prior degree of information borrowing across patient subgroups. The correlation parameters in BiEXNEX (i.e. ρ in the EX component and κ in the NEX component) quantify the association between the *subtrial-level* treatment effects, $\left(\theta_{k}^{t},\theta_{k}^{e}\right)$ , across the two endpoints. They do not represent within-patient correlation between toxicity and efficacy outcomes. In our data models to accommodate the observable, patient outcomes are linked to $\left(\theta_{k}^{t},\theta_{k}^{e}\right)$ through endpoint-specific likelihoods, and the joint modelling target is the bivariate distribution of treatment effects across subtrials. Modelling within-patient dependence would require specifying an additional joint patient-level structure (and additional parameters) which is typically weakly identified under limited phase II basket sample sizes; we therefore focus on treatment-effect correlation, which is most relevant for borrowing and our joint Go/no-Go decisions in Section 2.4.

In the NEX part, the mean $(m_{1k},m_{2k}{)}^{T}$ and standard deviation $(s_{1k},s_{2k}{)}^{T}$ for subtrial k are fixed and elicited before applying the method to trial data, except for the correlation κ uniformly distributing between −1 and 1. The NEX distribution is sometimes introduced as the ‘operational prior’ which are calibrated to accommodate considerable variability in the toxicity and efficacy effects across subtrials. Such operational priors are often vague or weakly informative.

The BiEXNEX model shows its simplicity as a bivariate analysis method with only two mixture components to estimate the subtrial-specific parameters. Yet it could be limited for circumstances when either toxicity or efficacy parameters only are exchangeable across subtrials, which we refer to as ‘asynchronous exchangeability’.

### 2.3. Extend BiEXNEX (E-BiEXNEX) model

We relax the assumption of simultaneously exchangeable treatment effects, and propose a more flexible model to extend BiEXNEX method, hereafter referred to as E-BiEXNEX. The E-BiEXNEX model for subtrial k is structured with four mixture components to accommodate asynchronous exchangeability, coupled with four sets of prior probabilities of exchangeability: with probability $\lambda_{1k}$ , both $\theta_{k}^{t}$ and $\theta_{k}^{e}$ are exchangeable across subtrials,

(4a) $$\left(\begin{array}{c} \theta_{k}^{t}\\ \theta_{k}^{e} \end{array}\right)|,\left(\begin{array}{c} \beta_{1}\\ \beta_{2} \end{array}\right),\left(\begin{array}{c} \phi_{1}\\ \phi_{2} \end{array}\right),\rho ∼N\left(\left(\begin{array}{c} \beta_{1}\\ \beta_{2} \end{array}\right),\left(\begin{array}{cc} \phi_{1}^{2} & \rho \phi_{1}\phi_{2}\\ \rho \phi_{1}\phi_{2} & \phi_{2}^{2} \end{array}\right)\right)$$

with probability $\lambda_{2k}$ , only $\theta_{k}^{t}$ is exchangeable,

(4b) $$\left(\begin{array}{c} \theta_{k}^{t}\\ \theta_{k}^{e} \end{array}\right)|\beta_{1},\phi_{1}∼N\left(\left(\begin{array}{c} \beta_{1}\\ m_{2k} \end{array}\right),\left(\begin{array}{cc} \phi_{1}^{2} & 0\\ 0 & s_{2k}^{2} \end{array}\right)\right)$$

with probability $\lambda_{3k}$ , only $\theta_{k}^{e}$ is exchangeable,

(4c) $$\left(\begin{array}{c} \theta_{k}^{t}\\ \theta_{k}^{e} \end{array}\right)|\beta_{2},\phi_{2}∼N\left(\left(\begin{array}{c} m_{1k}\\ \beta_{2} \end{array}\right),\left(\begin{array}{cc} s_{1k}^{2} & 0\\ 0 & \phi_{2}^{2} \end{array}\right)\right)$$

with probability $\lambda_{4k}$ , neither $\theta_{k}^{t}$ nor $\theta_{k}^{e}$ is exchangeable,

(4d) $$\left(\begin{array}{c} \theta_{k}^{t}\\ \theta_{k}^{e} \end{array}\right)|\kappa \;∼\;N\left(\left(\begin{array}{c} m_{1k}\\ m_{2k} \end{array}\right),\left(\begin{array}{cc} s_{1k}^{2} & \kappa s_{1k}s_{2k}\\ \kappa s_{1k}s_{2k} & s_{2k}^{2} \end{array}\right)\right)$$

where $\Sigma_{i=1}^{4}\lambda_{ik}=1$ and $0⩽\lambda_{ik}⩽1$ for each subtrial k . Similar to the parameter elaboration in the BiEXNEX model, $(\beta_{1},\beta_{2}{)}^{T}$ and $(\phi_{1},\phi_{2}{)}^{T}$ are unspecified and shared variables to allow for information borrowing among all subtrials, while $(m_{1k},m_{2k}{)}^{T}$ and $(s_{1k},s_{2k}{)}^{T}$ specified in advance are fixed and for subtrial k . The prior specifications for (hyper)parameters in E-BiEXNEX model will stay the same as those in the BiEXNEX modelling.

In components (4b) and (4c), only one endpoint is exchangeable across subtrials. We set the off-diagonal covariance to zero in these two mixed components as a parsimonious default, because introducing separate correlation parameters for each mixed component can be very weakly inferred with limited sample sizes and may lead to over-parameterisation. Importantly, this restriction does not remove dependence between toxicity and efficacy treatment effects overall: correlation is still (partly) accommodated through the full EX component (4a) and the full NEX component (4d), and through the mixture structure. A sample sensitivity analysis showing the justification of such correlation structure is attached to the Supplemental Material (Supplemental Appendix H).

The E-BiEXNEX model contains more configurations of the exchangeability and non-exchangeability distributions for toxicity and efficacy effects. We thus expect this model to be more robust than the BiEXNEX model, which would facilitate the inferences in situations where the degrees of information borrowing differ across the toxicity and efficacy treatment effects. For the E-BiEXNEX model, calculating posterior probabilities of ( $\lambda_{1k}+\lambda_{2k}$ ) can approximate the posterior probability of exchangeability marginally for the toxicity effects, and likewise, posterior probabilities of ( $\lambda_{1k}+\lambda_{3k}$ ) approximate that for the efficacy effects. Supplemental Appendix A in the Supplemental Material provides proof of such approximation, for which we re-express the E-BiEXNEX model using ancillary categorical variables and conditional probabilities.

Though the BiEXNEX and E-BiEXNEX models are designed for basket trials with multiple subtrials, the idea can be straightforwardly applicable to single-arm settings by cancelling the parameters with respect to control groups. The modelling details can be checked in the Supplemental Material (Supplemental Appendix B).

### 2.4. Decision-making rule

On the completion of a basket trial, data are analysed to yield Go or No-go recommendations towards a confirmatory phase III study. Two decision criteria are elaborated below for joint evaluation of $\theta_{k}^{t}$ and $\theta_{k}^{e}$ , where $\theta_{k}^{t}$ is log-odds ratio comparing the treatment against control for toxicity effect and $\theta_{k}^{e}$ is the difference of normal means between the treatment and control.

Firstly, a Go decision will only be taken for subtrial k if both (marginal) toxicity and efficacy constraints are satisfied as follows:

$$\text{Pr}(\theta_{k}^{t}<\delta_{0}|D)>\eta_{1}\;\text{and}\;\text{Pr}(\theta_{k}^{e}>\delta |D)>\eta_{2}$$

where D denotes the toxicity and efficacy data of all subtrials k=1,…,K , $\eta_{1}$ and $\eta_{2}$ are probability thresholds close to 1 and δ is the expected efficacy difference from the trial specialists. Similarly, $\delta_{0}$ is typically a clinically meaningful magnitude required to demonstrate that the treatment is safer than control, which could be determined by subject-matter experts. For illustration, we set the threshold to 0 for illustration meaning we expect to identify the treatment is no worse than control in terms of toxicity. Figure 2 illustrates the posterior interval probabilities involved in this decision criterion.

**Figure 2.**
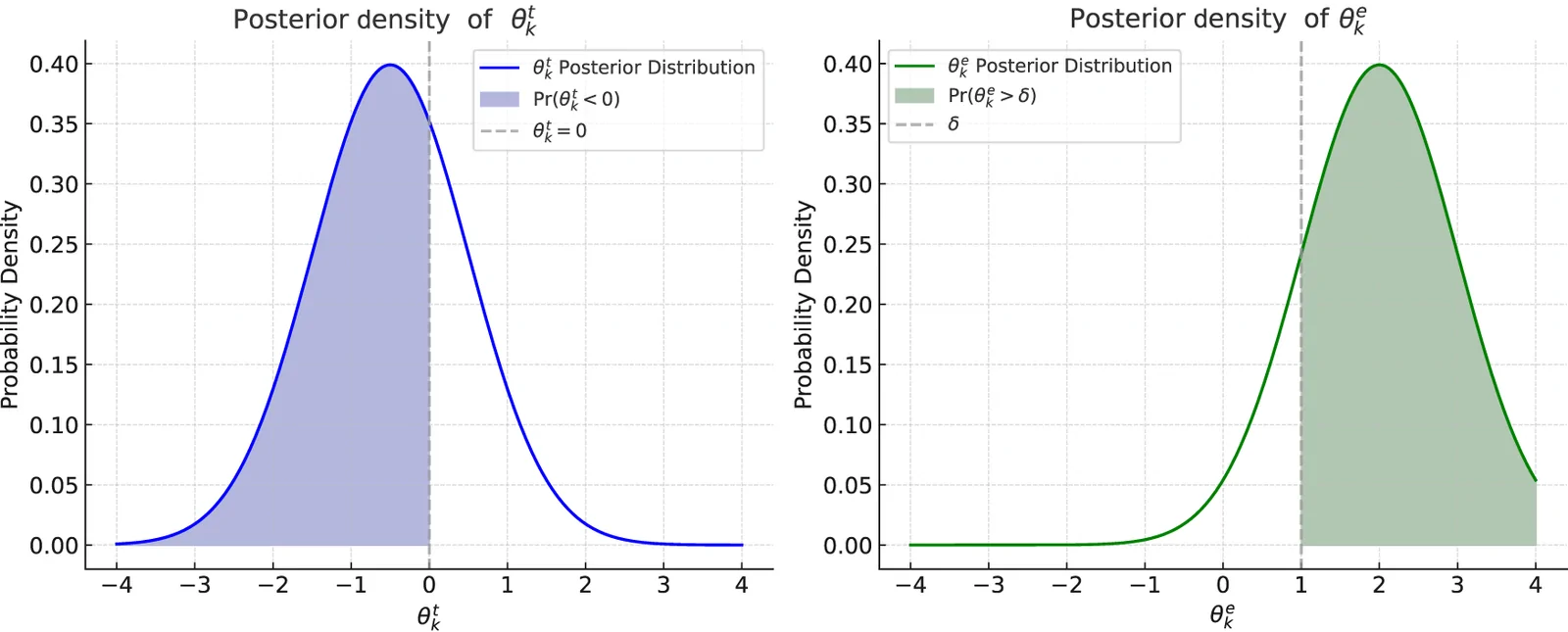
An example of the separate effect rule. The desirable treatment should have the area of light blue and green shaded parts greater than $\eta_{1}$ and $\eta_{2}$ , respectively.

This rule aims to make Go decisions only if we have enough belief (posterior probability bigger than a threshold) for toxicity and efficacy separately, which can be regarded as a combination of two marginal rules. However, this decision procedure does not completely conform to our objective of jointly evaluating treatment effects and modelling their possible correlations.

We then propose a joint decision rule involving correlations and only one probability cut-off:

$$\text{Pr}(\theta_{k}^{t}<\delta_{0}\text{ and }\theta_{k}^{e}>\delta |D)>\eta$$

where $\left(\theta_{k}^{t},\theta_{k}^{e}\right)$ is assessed in combination to include their correlation. Figure 3 presents an example of the joint rule in a decision-making procedure where $\left(\delta_{0},\delta )=(0,\delta \right)$ .

**Figure 3.**
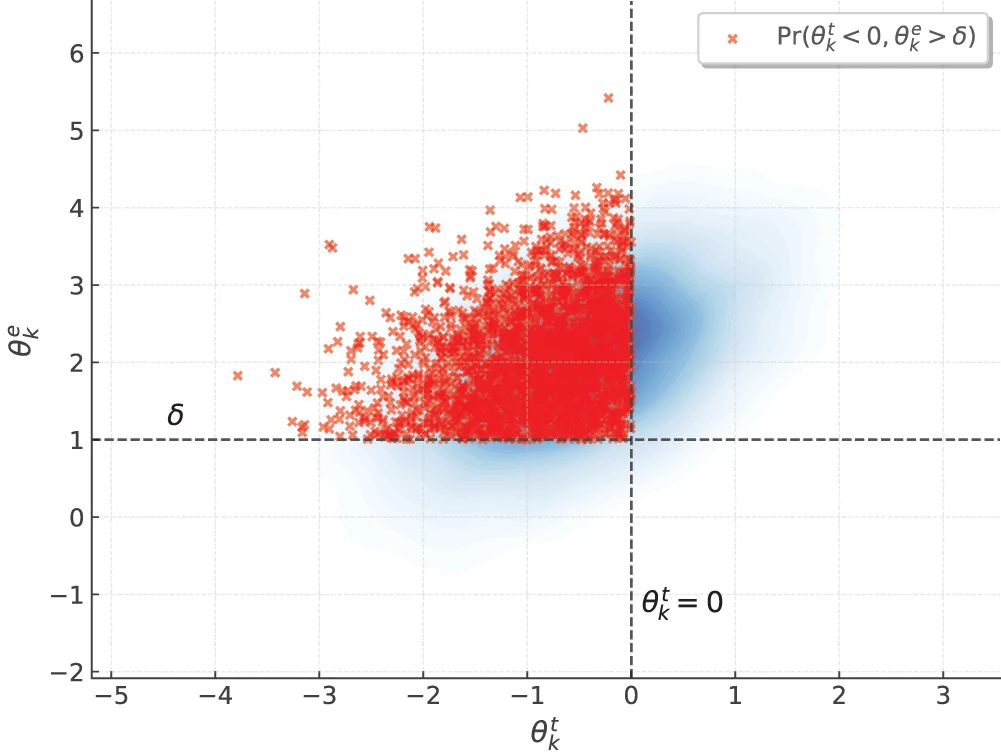
An example of the joint decision rule. The blue shaded area is the probability density of $\left(\theta_{k}^{t},\theta_{k}^{e}\right)$ . For the desirable treatment, we aim to find the proportion of red crosses in all posterior samples of $\left(\theta_{k}^{t},\theta_{k}^{e}\right)$ bigger than η .

Normally, the second decision rule could be rewritten into a more generalised form:

$$\text{Pr}\left((\theta_{k}^{t},\theta_{k}^{e})\in \Theta |D\right)>\eta$$

where Θ is a clinician-defined ‘desirable region’ that can explicitly encode trade-off between toxicity and efficacy (e.g. allowing higher toxicity only when efficacy is sufficiently large). The proposed second decision rule is achieved by setting Θ as all points located top-left of $\left(\delta_{0},\delta \right)$ at the $\left(\theta_{k}^{t},\theta_{k}^{e}\right)$ plane. In practice, clinicians may pursue a trade-off between $\theta_{k}^{t}$ and $\theta_{k}^{e}$ by adding some interdependent relationship to make more informed decisions. For example, a utility function can be formed as a weighted linear combination of $\theta_{k}^{t}$ and $\theta_{k}^{e}$ : $U(\theta_{k}^{t},\theta_{k}^{e}):=\omega_{U1}\times (-\theta_{k}^{t})+\omega_{U2}\times \theta_{k}^{e}$ ( $\omega_{U1}$ and $\omega_{U2}$ are pre-specified weights determining how much $-\theta_{k}^{t}$ and $\theta_{k}^{e}$ will contribute to the final utility). Then the generalised decision rule becomes the following equation:

$$\text{Pr}\left(U(\theta_{k}^{t},\theta_{k}^{e})>U_{0}|D\right)>\eta$$

where $U_{0}$ is the minimally expected utility we would like to obtain, pre-fixed in the trial protocol. In such contexts, the desirable region $\Theta =\{(\theta_{k}^{t},\theta_{k}^{e})|U(\theta_{k}^{t},\theta_{k}^{e})>U_{0}\}$ . Additionally, Θ can be established by a series of manually indicated contour lines, like what^28^ had done.

## 3. Simulation study

In this section, we evaluate the operating characteristics of basket trials analysed using the proposed Bayesian models with simulations. We simulate basket trial data with K=6 subtrials with the data-generation mechanism described in Section 3.1 and simulation scenarios elaborated in Section 3.2.

### 3.1. Data-generating mechanism

Based on Mozgunov et al.,^29^ we design a data-generation mechanism to simulate correlated observations of the toxicity and efficacy data from the same individual. This underpins the potential correlation between treatment effects $\theta_{k}^{t}$ and $\theta_{k}^{e}$ within each subtrial k . This data-generation mechanism is elaborated with three steps below. It should be noted that the data-generation algorithm concentrates on how to generate (correlated) patient outcomes under prespecified fixed true treatment effects ( $\theta_{k}^{t}$ and $\theta_{k}^{e}$ ) for every subtrial. All constants mentioned in the following mechanism will not impact final model comparisons as the additive constants will be cancelled by taking differences between means, and the covariance matrices are identical for each model when generating sample data.

1. Generate bivariate patient-level data for the control group (*C*) in subtrial k : for patient i , generate $\left(x_{iCk},z_{iCk}{)}^{T}∼N(\mu_{Ck}^{\prime },\Sigma_{Ck}^{\prime }\right)$ , where $x_{iCk}$ is a latent variable for generating toxicity outcome, and $z_{iCk}$ is the efficacy observation, $\mu_{Ck}^{\prime }=(\mu_{1Ck}^{\prime },\mu_{2Ck}^{\prime }{)}^{T}$ and $\Sigma_{Ck}^{\prime }$ symbolise the mean and covariance matrix. In this simulation study, we set $\mu_{1C1}^{\prime },\ldots ,\mu_{1C6}^{\prime }=(1.2,1.5,1.02,0.88,1.05,0.96)$ , $\mu_{2Ck}^{\prime }=\mu_{1Ck}^{\prime }+2.0$ , and $\Sigma_{Ck}^{\prime }=\left(\begin{array}{cc} \sigma_{1Ck}^{2} & \rho^{\prime }\sigma_{1Ck}\sigma_{2Ck}\\ \rho^{\prime }\sigma_{1Ck}\sigma_{2Ck} & \sigma_{2Ck}^{2} \end{array}\right)=\left(\begin{array}{cc} 1^{2} & 0.8\times 1\times 1\\ 0.8\times 1\times 1 & 1^{2} \end{array}\right)$ for each k .
2. Dichotomise $x_{iCk}$ : Given a fixed threshold L , let patient-level toxicity outcomes $y_{iCk}=\text{I}(x_{iCk}⩽L)$ such that the binary structure is observable, where I( ⋅ ) is an indicator function =1 for toxicity and 0 for no toxicity. It should be noted that $\mu_{2Ck}^{\prime }$ is the same as $\mu_{Ck}$ in the efficacy observation model described in Section 2.1. L is set to 0.8 in this simulation study.
3. Data generation for treatment groups: Suppose we have known $\theta_{k}^{t}$ and $\theta_{k}^{e}$ for each subtrial k , the mean vector $\mu_{Ek}^{\prime }=(\mu_{1Ek}^{\prime },\mu_{2Ek}^{\prime })$ can be easily computed via the observation models. We have the identity link for average efficacy responses, hence $\mu_{2Ek}^{\prime }=\mu_{2Ck}^{\prime }+\theta_{k}^{e}$ ( $=\mu_{Ek}$ in Section 2.1). For obtaining $\mu_{1Ek}^{\prime }$ , we can approximate it by using numerical methods. First, the theoretical toxicity response rate in the treatment group (*E*) can be computed as follows:
$$\begin{array}{rl} p_{Ek}=\text{Pr}(x_{iEk}⩽L) & =\Phi (L;\mu_{1Ek}^{\prime },\sigma_{1Ek})\\ & =1-\Phi (\mu_{1Ek}^{\prime };L,\sigma_{1Ek}) \end{array}$$
  where $x_{iEk}$ is the patient-level latent variable of toxicity in treatment groups, assumed to follow a normal distribution with mean $\mu_{1Ek}^{\prime }$ and standard deviation $\sigma_{1Ek}$ ; and Φ(x;a,b) is the cumulative distribution function (CDF) of $N(a,b^{2})$ at x . Therefore,
(5) $$\mu_{1Ek}^{\prime }=\Phi^{-1}(1-p_{Ek};L,\sigma_{1Ek})$$
  where $\Phi^{-1}(\cdot )$ is the inverse function of Φ(⋅) , which explicitly exists and is unique because Φ(⋅) is a strictly monotonic function on the real line. Fixed $\sigma_{1Ek}$ at 1, we only need to derive the value of $p_{Ek}$ to attain $\mu_{1Ek}^{\prime }$ . Likewise, the toxicity response rate in the control group
$$p_{Ck}=\text{Pr}(x_{iCk}⩽L)=\Phi (L;\mu_{1Ck}^{\prime },\sigma_{1Ck})$$
  where $\mu_{1Ck}^{\prime }$ and $\sigma_{1Ck}(=1)$ have been discussed in step 1. Applying the logit link for toxicity, we have
$$p_{Ek}={\text{logit}}^{-1}(\text{logit}(p_{Ck})+\theta_{k}^{t})$$
  Then $\mu_{1Ek}^{\prime }$ can be obtained via equation (5). Now pseudo-observations for treatment groups can be generated from $\left(x_{iEk},z_{iEk}{)}^{T}∼N(\mu_{Ek}^{\prime },\Sigma_{Ek}^{\prime }\right)$ , with $\mu_{Ek}^{\prime }=(\mu_{1Ek}^{\prime },\mu_{2Ek}^{\prime }{)}^{T}$ and
$$\begin{array}{rl} \Sigma_{Ek}^{\prime } & =\left(\begin{array}{cc} \sigma_{1Ek}^{2} & \rho^{\prime \prime }\sigma_{1Ek}\sigma_{2Ek}\\ \rho^{\prime \prime }\sigma_{1Ek}\sigma_{2Ek} & \sigma_{2Ek}^{2} \end{array}\right)\\ & =\left(\begin{array}{cc} 1^{2} & 0.8\times 1\times 1\\ 0.8\times 1\times 1 & 1^{2} \end{array}\right) \end{array}$$
  The continuous efficacy and binary toxicity outcomes on the treatment group across subtrials will be $z_{iEk}$ and $y_{iEk}=\text{I}(x_{iEk}⩽L)$ , respectively.

An important property of the trial outcomes is the induced correlation between the binomial toxicity and continuous efficacy endpoints.^30,31^ Our data-generation steps naturally harbour patient-level correlations within same subtrial k and treatment group j :

$$\begin{array}{rl} \text{Cor}(y_{ijk},z_{ijk}) & =\frac{E(y_{ijk}z_{ijk})-E(y_{ijk})E(z_{ijk})}{\sqrt{\text{Var}(y_{ijk})}\sqrt{\text{Var}(z_{ijk})}}\\ & =\frac{1}{\sigma_{2jk}}\frac{E(y_{ijk}z_{ijk})-\mu_{2jk}^{\prime }\Phi (L;\mu_{1jk}^{\prime },\sigma_{1jk})}{\sqrt{\Phi (L;\mu_{1jk}^{\prime },\sigma_{1jk})-\Phi (L;\mu_{1jk}^{\prime },\sigma_{1jk}{)}^{2}}} \end{array}$$

where

$$E(y_{ijk}z_{ijk})=\iint_{R^{2}}zI(x⩽L)\phi \{(x,z{)}^{T};\mu_{jk}^{\prime },\Sigma_{jk}^{\prime }\}dxdz$$

and ϕ(⋅) is the probability density function of $N(\mu_{jk}^{\prime },\Sigma_{jk}^{\prime })$ . Therefore, the correlation between outcomes can be partly controlled by adjusting the threshold L , but the detailed adjustment is beyond the scope of this article and hence will not be discussed.

### 3.2. Basic settings

We simulate the basket trial data assuming equal randomisation between E and C within each subtrial k=1,…,6 . The subtrial sample sizes are set as: $N_{k}=\{20,20,20,12,24,20\}$ with $n_{Ck}=n_{Ek}=\frac{1}{2}N_{k}$ . We compare our analysis models with their alternatives as follows:

- BHM: Standard Bayesian hierarchical model, equivalent to setting each $\omega_{k}=1$ in BiEXNEX.
- IndEXNEX: Independent EXNEX models fitted to toxicity and efficacy data. This is a special version of our proposed E-BiEXNEX method without correlations.
- SA: stand-alone analysis. We set $\omega_{k}=0$ for all k=1,…,K (no information borrowing across subtrials) and, as a simple working-independence baseline, we also set the correlation between $\left(\theta_{k}^{t},\theta_{k}^{e}\right)$ to 0. This represents analysing toxicity and efficacy separately within each subtrial.

The comparison will be made using Bayesian analogues of the frequentist operating characteristics such as type I error rates and statistical power. This can provide consistent evidence as exploiting the frequentists’ approaches, and simplifying complex trial designs to facilitate regulatory compliance^32^:

- (Bayesian) Type I error: for subtrial k , the probability that an erroneous Go decision is given under no efficacy or high toxicity.
- Power: for subtrial k , the probability that a Go decision is recommended with the truly safer and efficacious treatment.

We consider the following null and alternative scenarios for evaluating the type I error and power for each modelling approach. These scenarios are specified to reflect small to large between-subgroup heterogeneity, as well as low or high correlation between the toxicity and efficacy effects.

#### 3.2.1. Null scenarios

Table 1 lists a broad range of scenarios with global and mixed null settings for explorations of type I error rates using the bivariate analysis models.

**Table 1.** Null scenarios defined by the subtrial-specific toxicity and efficacy treatment effects.

		Subtrial k						
Scenario		1 (10)	2 (10)	3 (10)	4 (6)	5 (12)	6 (10)	Description
Global Null	$\theta_{k}^{t}$	0	0	0	0	0	0	$\theta_{k}^{t}$ and $\theta_{k}^{e}$ are both 0 for all subtrials
	$\theta_{k}^{e}$	0	0	0	0	0	0	
1a	$\theta_{k}^{t}$	0	0	0	0	0	0	$\theta_{k}^{e}=0.75$ across all k for much borrowing
	$\theta_{k}^{e}$	0.75	0.75	0.75	0.75	0.75	0.75	
1b	$\theta_{k}^{t}$	0	0	0	0	0	0	$\theta_{k}^{e}=2.25$ across all k for much borrowing
	$\theta_{k}^{e}$	2.25	2.25	2.25	2.25	2.25	2.25	
1c	$\theta_{k}^{t}$	0	0	0	0	0	0	$\theta_{k}^{e}$ varies largely
	$\theta_{k}^{e}$	0	0.75	1.75	4	8	12	
2a	$\theta_{k}^{t}$	−1.25	−1.25	−1.25	−1.25	−1.25	−1.25	$\theta_{k}^{t}=-1.25$ across all k for much borrowing
	$\theta_{k}^{e}$	0	0	0	0	0	0	
2b	$\theta_{k}^{t}$	−2.5	−2.5	−2.5	−2.5	−2.5	−2.5	$\theta_{k}^{t}=-2.5$ across all k for much borrowing
	$\theta_{k}^{e}$	0	0	0	0	0	0	
2c	$\theta_{k}^{t}$	0	−0.55	−1.55	−3.1	−6.6	−9.5	$\theta_{k}^{t}$ varies considerably
	$\theta_{k}^{e}$	0	0	0	0	0	0	

Number in the parentheses is $n_{Ck}=n_{Ek}$ , so the total subtrial sample size is twice the number.

Based on the above null settings, we consider the borrowing of information for toxicity or efficacy effects solely or both to investigate type I error rates under different situations, so that the operating characteristics of our model can be thoroughly appraised.

#### 3.2.2. Scenarios for power calculation

Six simulation scenarios are presented in Table 2 for power calculation. As a succinct summary, the total six scenarios can be roughly divided into three groups:

**Table 2.** Six additional scenarios for power calculation.

		Subtrial k with (half-)sample size $n_{Ck}=n_{Ek}$							
Scenario		1 (10)	2 (10)	3 (10)	4 (6)	5 (12)	6 (10)	SD	Description
Ia	$\theta_{k}^{t}$	−0.8	−0.68	−0.72	−0.77	−0.96	−0.92	0.11	$\theta_{k}^{t}$ and $\theta_{k}^{e}$ are similar across subtrials
	$\theta_{k}^{e}$	0.76	0.82	0.69	0.72	0.75	0.66	0.06	
Ib	$\theta_{k}^{t}$	−0.95	−0.82	−1.07	0.20	−1.67	−2.10	0.79	$\theta_{k}^{t}$ and $\theta_{k}^{e}$ are partly similar across subtrials
	$\theta_{k}^{e}$	0.77	0.73	0.80	1.80	−0.46	−1.20	1.06	
IIa	$\theta_{k}^{t}$	−1.4	1.4	0.87	−2.2	0.15	−0.8	1.38	$\theta_{k}^{t}$ and $\theta_{k}^{e}$ are largely different, with low correlation (−0.18)
	$\theta_{k}^{e}$	−1.77	0.05	0.68	1.80	−0.46	2.12	1.45	
IIb	$\theta_{k}^{t}$	−1.4	1.4	−0.87	−2.2	0.15	0.8	1.37	$\theta_{k}^{t}$ and $\theta_{k}^{e}$ are largely different, with high correlation (−0.94)
	$\theta_{k}^{e}$	1.43	−1.66	1.08	1.46	−0.02	−1.84	1.51	
IIIa	$\theta_{k}^{t}$	−1.5	2.34	0.87	−0.55	−3	4.22	2.63	$\theta_{k}^{e}$ is similar, but $\theta_{k}^{t}$ is not
	$\theta_{k}^{e}$	1.0	1.0	1.0	1.0	1.0	1.0	0	
IIIb	$\theta_{k}^{t}$	−1.2	−1.2	−1.2	−1.2	−1.2	−1.2	0	$\theta_{k}^{t}$ is similar, but $\theta_{k}^{e}$ is not
	$\theta_{k}^{e}$	−1.27	3.48	2.78	−0.02	1.15	−0.12	1.83	

The first row indicates toxicity treatment effects, the second row shows efficacy treatment effects, and the last column provides simple descriptions. The middle column SD gives the standard deviation of treatment effects accordingly.

- Treatment effects $\theta_{k}^{t}$ and $\theta_{k}^{e}$ are similar or partly similar: scenarios Ia and Ib.
-
$\theta_{k}^{t}$ and $\theta_{k}^{e}$ are largely different: scenario IIa (correlation as low as −0.18 between $\theta_{1}^{t},\theta_{2}^{t},\ldots ,\theta_{K}^{t}$ and $\theta_{1}^{e},\theta_{2}^{e},\ldots ,\theta_{K}^{e}$ ) and scenario IIb (high negative correlation as −0.94).
- One of $\theta_{k}^{t}$ and $\theta_{k}^{e}$ is similar while the other is not: scenarios IIIa and IIIb.

where the similarity measure of treatment effects is their standard deviation (SD), that is, SD > 1 means the effects are considered largely different. We include the accordingly transformed toxicity response rates and mean efficacy for treatment and control groups in the Supplemental Material (Supplemental Appendix C) for the reference.

### 3.3. Prior specification

We assume no specific opinion on exchangeability and non-exchangeability for toxicity and efficacy treatment effects, so all related prior probabilities of exchangeability would be set to $\omega_{k}=0.5$ in BiEXNEX, and $\lambda_{ik}=0.25$ in E-BiEXNEX for each of the four components and subtrial k . In order to make models comparable, the identical priors of corresponding parameters for each simulated method will be employed. For the observation model, we assume $\alpha_{k}^{t}$ and $\alpha_{k}^{e}$ follow a uninformative prior $N(0,10^{2})$ for all subtrials, which can accommodate a large range of possible baseline effects, together with a vague variance prior of efficacy observations $\sigma_{jk}^{2}∼\text{Half-}N(5^{2})$ , where Half-N(x) means the half-normal distribution with variance x folded at mean 0.

Vague priors are placed on $\beta_{1}$ and $\beta_{2}$ : $\beta_{1},\beta_{2}∼N(0,5^{2})$ . We set $\phi_{1}^{2},\phi_{2}^{2}∼\text{Half-}N(0.5^{2})$ because of the need to constrain the upper value of such variances when clinically relevant differences are assumed to be exchangeable, and the scale parameter 0.5 was chosen to allow medium to substantial between-subtrial heterogeneity.^8^ On the other hand, subtrial specific parameters $m_{1k},m_{2k}=0$ and $s_{1k},s_{2k}=5$ for all subtrials, and the unknown correlations ρ,κ are presupposed to follow a flat prior U(−1,1) .

We use Markov Chain Monte Carlo (MCMC) method to simulate each model (not generate the data) and record the posterior samples of treatment effects, in which the median is adopted as the point estimate as it is robust to skewed posterior densities. To control the Monte Carlo standard error under 0.5%, we perform $10^{4}$ repetitions in the study^33^ for each model and scenario on a computer with R4.3.2 and Just Another Gibbs Sampler (JAGS4.3.1). We have four MCMC chains for every model in a single repetition, each with 2000 burn-in samples and $10^{4}$ iterations.

### 3.4. Results

All figures and main results in this section are presented by applying the joint decision rule (Section 2.4), because of negligible difference between the simulated results by both rules. We emphasise that the following power values reflect the specific illustrative design settings considered here, including small phase II subtrial sample sizes and a joint Go decision requiring both acceptable toxicity and promising efficacy. Our main objective is comparative performance across methods under the same design constraints, under which absolute Go probabilities can be modest even when true effects are present.

Figures 4 shows the type I error rates for all models under various null scenarios. Under the global null scenario (top left panel of Figure 4), all methods, including BHM, BiEXNEX, E-BiEXNEX, IndEXNEX and SA, demonstrate small and comparable type I error rates. This indicates that, where there is no toxicity and efficacy difference between the treatment and control group, all approaches perform similarly well with low error rates.

**Figure 4.**
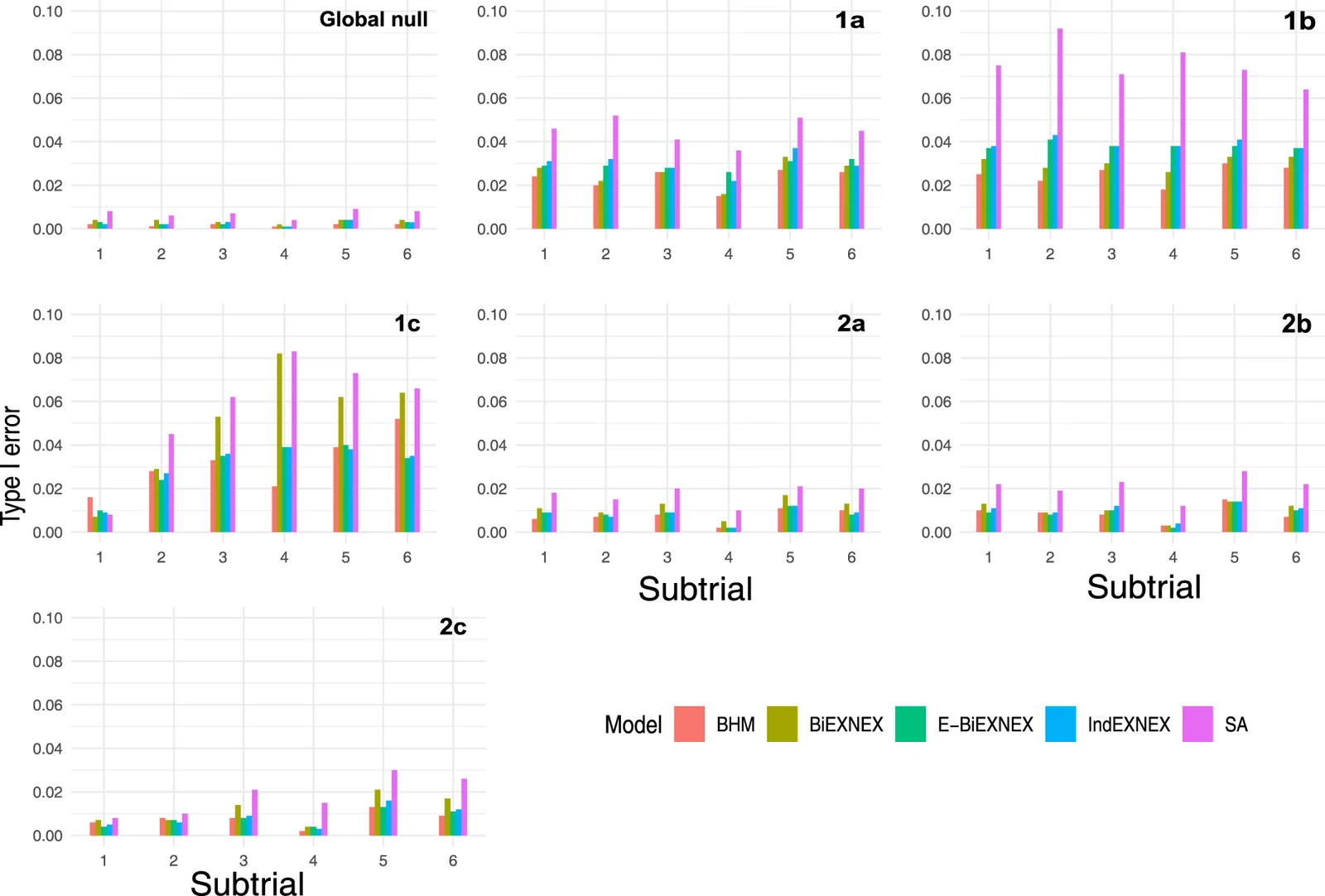
Bayesian analogues of type I error rates for each null scenario in Section 3.2.1. The *x*-axis is subtrial index and *y*-axis is type I error rate for each model.

In the mixed null scenarios 1a–1c, where $\theta_{k}^{t}=0$ but $\theta_{k}^{e}$ increases (from 1a to 1b) or varies (1c), noticeable differences emerge. The stand-alone analysis (SA) method consistently produces the highest type I error rates compared with approaches borrowing information, because it generates wider posterior distributions of treatment effects without borrowing information across patient subgroups, causing higher type I error rates than other methods exploiting more information. Although BHM and BiEXNEX model perform adequately well when both toxicity and efficacy treatment effects are exchangeable, their type I error rates can fluctuate to a significant extent when only one effect is exchangeable. The two models perform well in scenarios 1a and 1b due to their capability to correctly specify the substantial degree of information borrowing due to identical $\theta_{k}^{t}$ and $\theta_{k}^{e}$ across all subtrials. While BHM and BiEXNEX produce largely varied type I error in scenario 1c, where only $\theta_{k}^{t}$ is exchangeable, since they cannot accommodate the different exchangeability of treatment effects. To be exact, in scenario 1c, the BHM method performs well in subtrials 1–4 but generate rapidly increasing error in subtrials 5 and 6 as it borrows too much information from subtrials 1 to 4 which have more similar $\theta_{k}^{e}$ in contrast with subtrials 5 and 6. The BiEXNEX model produces larger error rates as it cannot specify the correct pattern of information borrowing, but the type I error of BiEXNEX is still less than SA (it is natural for BiEXNEX as a combination of SA and BHM). We also examined whether allowing between-subtrial correlation in SA could explain its higher type I error in scenarios 1a–1c. A sensitivity analysis in which we compared Go decision rates between original SA and SA retained correlation between $\left(\theta_{k}^{t},\theta_{k}^{e}\right)$ suggested adding more correlation parameters would not reduce type I error (see Supplemental Appendix I). This suggests that the inflation of error rate is primarily driven by the absence of borrowing across subtrials under small sample sizes rather than by the working-independence assumption.

In scenarios 2a–2c, the SA method again exhibits the largest type I error rates compared with other models. The reason is that, for scenarios 2a to 2c, the information borrowing should be encouraged, leading to smaller false positive rates of methods which enable such an attribute (BiEXNEX, E-BiEXNEX and IndEXNEX). Scenarios 2a and 2b are natural to borrow because they have the same $\theta_{k}^{t}$ and $\theta_{k}^{e}$ across all subtrials. Scenario 2c also implies borrowing though subtrials have substantially varied $\theta_{k}^{t}$ which are saturated on the logit scale. That is, extremely negative $\theta_{k}^{t}$ give rise to alike $p_{Ek}$ after the logit transformation in our data-generation mechanism. For example, if $p_{Ck}=0.3$ and $\theta_{k}^{t}=-3.1,-6.6,-9.5$ , $p_{Ek}={\text{logit}}^{-1}(\text{logit}(p_{Ck})+\theta_{k}^{t})\approx 0.02,0.0006,0.00003$ which will have approximately identical effects in data generation under the current limited sample size. Consequently, the shared information degree is high in scenario 2c. Besides, the probability of making erroneous decisions for at least one subtrial (defined as overall error rate, OER) is shown in the Supplemental Material (Appendix D) as a reference operating characteristic when the ‘family-wise error rates’ are considered.

Figure 5 illustrates the statistical power for all models under each scenario. With differing levels of similarities between treatment effects, all approaches have different efficiency and robustness. When the dissimilarity of treatment effects grows (e.g. from scenarios Ia to IIa), the BHM performs more inefficient while SA produces larger power. In scenario Ia, where treatment effects are similar across subtrials, the BHM method achieves the highest power. This performance is expected due to the effective information sharing enabled by the small variability in treatment effects. However, in scenarios IIa and IIb, the performance of BHM is unsatisfactory because of the excessive borrowing of information from considerably varied treatment effects. By contrast, SA, which does not borrow information across subtrials, shows the opposite pattern which goes from one extreme to the other. Other methods are able to produce higher power in scenarios Ia, Ib, IIa and IIb as they consider the possibility of the high level of information borrowing or non-exchangeable treatment effects.

**Figure 5.**
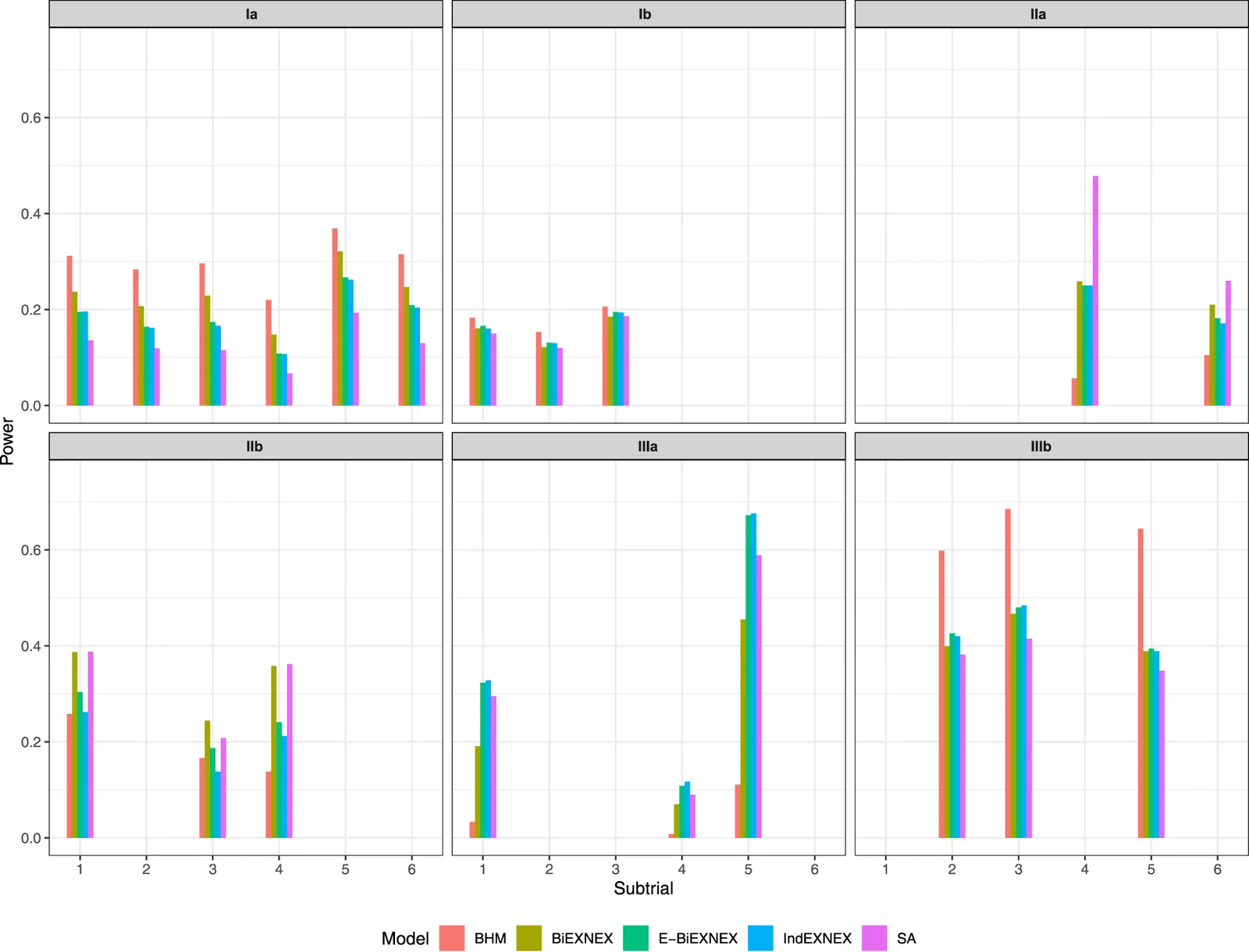
Bayesian analogues of power for each scenario, model and subtrial. The Go decision proportions under the subtrials with overly toxicity or inefficacy are erased from the figure.

The obvious correlation between treatment effects also has a noticeable influence on model performance, especially when both treatment effects vary substantially (scenarios IIa and IIb). It is clear that in scenario IIb where treatment effects are largely different with high correlation, BiEXNEX can perfectly specify the scenario, leading to very similar power in subtrials 1 and 4, and even higher power in subtrial 3, compared with SA which does not consider possible correlation. On average, E-BiEXNEX produces about 5% higher power than IndEXNEX in scenario IIb as it accommodates such a correlation.

In scenarios IIIa and IIIb where only $\theta_{k}^{t}$ or $\theta_{k}^{e}$ varies dramatically, BHM generates lower power than SA in IIIa but higher in IIIb under the current sample sizes, while E-BiEXNEX and IndEXNEX models produce similar power. In scenario IIIa, where $\theta_{k}^{e}$ is the same but $\theta_{k}^{t}$ changes significantly, E-BiEXNEX and IndEXNEX achieve higher power than BHM in the favourable subtrials, reflecting their ability to account for exchangeability in only one treatment effect. In comparison, BHM produces the lowest power, falling below that of SA. In scenario IIIb, where $\theta_{k}^{t}$ is identical and $\theta_{k}^{e}$ varies, BHM performs exceptionally well, achieving the highest power, while SA reports the lowest power, that is, below 0.4 in most cases. The reason lies in the current sample sizes, which can be sufficient to infer efficacy-related parameters from continuous data, but far not enough to obtain precise toxicity treatment effect for binary toxicity data. This aligns well with the statement by Altman and Royston^34^ that less information is carried in dichotomised data compared to continuous data. Therefore, in scenario IIIb, the same $\theta_{k}^{t}$ indicates borrowing of information could be recommended under the current sample size.

We also investigate the scenarios with only one exchangeable treatment effects with smaller sample sizes (half of the current sizes). The results show E-BiEXNEX achieves the highest power, regardless which treatment effects, on either toxicity or efficacy, are exchangeable, when the smaller samples cannot provide sufficient information even in continuous data. Relevant figures are included in the Supplemental Material (Supplemental Appendix E). For results obtained by applying the first decision criterion concerning marginal effects, we refer the interested readers to the Supplemental Material (Supplemental Appendix G) additionally.

Additionally, we investigate the impact of correlations between treatment effects on the posterior weights of our models that permit information borrowing. It was found that when $\theta_{k}^{t}$ and $\theta_{k}^{e}$ are highly correlated (e.g. the correlation is near −0.9) and largely different (so less information borrowing from their differences), the E-BiEXNEX model will give higher (3%–8%) posterior weight to ‘both treatment effects are non-exchangeable’ compared with using IndEXNEX. However, when $\theta_{k}^{t}$ and $\theta_{k}^{e}$ both are very similar, the correlation will have nearly no impact on the posterior weights. Therefore, the similarity between treatment effects is the main factor to influence the information borrowing weights, while the treatment effect correlation does have some impact on the posterior weight under low similarity and highly correlated effects scenarios.

To investigate how sample sizes can impact the absolute power shown in these results, we conducted an additional large-sample sensitivity analysis. We retained the original six power scenarios in Table 2 and the same joint decision rule, but increased the per-arm sample size to $n_{Ck}=n_{Ek}=50$ for each subtrial, and re-ran the simulations for all comparators: BHM, BiEXNEX, E-BiEXNEX, IndEXNEX and SA. As expected, increasing the sample size can improve power for all methods. Under such contexts, E-BiEXNEX could achieve over 70% power for most subtrials and scenarios. However, some difficult true-positive subtrials remained below, particularly when the true toxicity or efficacy effect was close to the joint decision boundary. These results indicate that larger sample sizes can improve absolute power, but that achieving conventional power levels remains scenario-dependent and clinically specific under the proposed joint toxicity–efficacy decision rule. Further details, such as power for all methods, can be viewed in the Supplemental Material.

The results highlight clear trade-offs between the models and the appealing robustness of E-BiEXNEX, which demonstrates steady performance across all scenarios, while achieving low type I error rates and competitive power relative to the alternative methods under the same simulation settings. SA, while providing larger type I error rates, performs acceptably in scenarios with substantial heterogeneity in treatment effects given the current large sample sizes. BHM excels when treatment effects are highly similar but struggles in scenarios with large variability in the toxicity and efficacy effects across subtrials. IndEXNEX achieves balanced performance, particularly in cases involving only one exchangeable effect, while it loses to E-BiEXNEX when varied treatment effects are highly correlated. The BiEXNEX method strikes a balance between BHM and SA, but it can also miss situations with solely exchangeable treatment effects. Overall, E-BiEXNEX is shown to be the most general method, delivering consistently small type I error rates and competitive power across all tested scenarios.

## 4. Discussion

Continued toxicity monitoring in phase II clinical trials gains increasing attention in drug development to recommend not only efficacious but safe treatments for further investigation. Basket trials spark natural possibilities of information borrowing across subtrials, establishing a joint analysis of efficacy and toxicity while accounting for patient heterogeneity in settings of precision medicine. In this article, we have proposed two bivariate Bayesian analysis models to this end, with a comprehensive simulation study conducted to evaluate the trial operating characteristics for each model and their alternatives.

From Section 3, BiEXNEX and E-BiEXNEX are more robust than BHM and SA across all scenarios, and E-BiEXNEX can flexibly accommodate scenarios in which only a single treatment effect is exchangeable, thus producing greater power. At the same time, small and stable type I error rates are achieved by E-BiEXNEX in all null scenarios. Correlation between treatment effects can have some impact on power calculation, but the similarity is the main factor. We conclude that when both treatment effects are assumed to be similar or largely different, BiEXNEX model is recommended to achieve higher power and safe type I error rates; when asynchronous exchangeability is expected or no prior knowledge suggesting so, E-BiEXNEX is a more appropriate choice for robust decision making.

Our proposed bivariate Bayesian analysis models can be relevant for settings with co-primary endpoints to claim efficacy. Investigators may follow our strategy to accommodate partial exchangeability of the subtrial-specific parameters. Additional consideration of incorporating known correlation structure, or assumptions of delayed response, can lead to valuable extension to the present work.

We would also note that the methodology allows alternative parameter models to be considered for cases where some subtrials have more similar treatment effects (captured by $\theta_{k}^{t}$ and $\theta_{k}^{e}$ ) between themselves than with others. Inspirations may be drawn from Zheng and Wason^10^ and Ouma et al.^35^ that enables pairwise borrowing of information, or Chen and Lee^11^ which involves clustering of subgroups for efficient information borrowing. Another research avenue to pursue is to develop sample size determination that accounts for the joint evaluation of efficacy and toxicity, with borrowing of information across subtrials.^36^ A practical implication of our simulation study is that, although information borrowing can improve power relative to non-borrowing approaches, it cannot fully compensate for limited within-subtrial information, especially when a Go decision requires simultaneous evidence of acceptable toxicity and promising efficacy. Therefore, in applications, the subtrial sample size and posterior probability thresholds should be prospectively calibrated to achieve prespecified operating characteristics, including acceptable subtrial-level power and type I error. Finally, we are interested in exploring the joint evaluation of toxicity and efficacy in basket trials where simultaneous dose-finding procedures may be implemented across patient subgroups. One possibility is to extend our Bayesian models to facilitate the estimation of dose–toxicity and dose–response curves in such settings, with the principal aim for identification of the optimal biological dose.^37^ Another direction in dose-finding settings is the consideration for late-onset toxicity which is common in clinical practice.^38^ Finally, if directly modelling multi-level toxicity probabilities is required, multinomial or ordinal models can be considered. To this research direction, how to address the excessive number of parameters involved in information borrowing would be another challenge for possible future work.

## Supplemental Material

sj-pdf-1-smm-10.1177_09622802261465336 - Supplemental material for Robust Bayesian hierarchical models for basket trials enabling joint evaluation of toxicity and efficacy Supplemental material, sj-pdf-1-smm-10.1177_09622802261465336 for Robust Bayesian hierarchical models for basket trials enabling joint evaluation of toxicity and efficacy by Zhi Cao, Pavel Mozgunov and Haiyan Zheng in Statistical Methods in Medical Research
