# Supplementary material for: Robust Bayesian hierarchical models for basket trials enabling joint evaluation of toxicity and efficacy
Source: Stat Methods Med Res. 2026 Sep 2;35(10):2061–75. doi: 10.1177/09622802261465336 (PMC13646420; doi:10.1177/09622802261465336)
Supplement: Supplementary file 1 [file sj-pdf-1-smm-10.1177_09622802261465336.pdf]

Supplementary Material

Another model stipulation for E-BiEXNEX

For subtrial k,

- $\mathcal{C}(\cdot)$: categorical distribution
- $\mathcal{N}(\cdot)$: two-dimensional normal distribution
- Z_{tk} or Z_{ek}: the exchangeability indicators as auxiliary variables, where t means toxicity and e denotes efficacy

$$\begin{aligned} \mathrm{Z}_{tk} &\sim \mathcal{C}(\boldsymbol{\pi}_{tk}) \\ \mathrm{Z}_{ek} &\sim \mathcal{C}(\boldsymbol{\pi}_{ek}) \\ \begin{pmatrix}\theta_k^t\\ \theta_k^e\end{pmatrix}\Bigg| \mathrm{Z}_{tk} = i, \mathrm{Z}_{ek} = j &\sim \mathcal{N}(\boldsymbol{\mu}_{ijk}, \boldsymbol{\Sigma}_{ijk}) \end{aligned} \tag{6}$$

where i and j can be 1 (exchangeable) or 2 (non-exchangeable); $\mathcal{N}(\cdot,\cdot)$ is the two-dimensional distribution with mean $\boldsymbol{\mu}_{ijk}$ and covariance matrix $\boldsymbol{\Sigma}_{ijk}$ which can represent the parameters of each component in the E-BiEXNEX (Section E-BiEXNEX model) model (i.e.

$$\begin{aligned} (\boldsymbol{\mu}_{11k}, \boldsymbol{\Sigma}_{11k}) &= \left[\begin{pmatrix}\beta_1\\ \beta_2\end{pmatrix}, \begin{pmatrix}\phi_1^2 & \rho\phi_1\phi_2\\ \rho\phi_1\phi_2 & \phi_2^2\end{pmatrix}\right] \\ (\boldsymbol{\mu}_{12k}, \boldsymbol{\Sigma}_{12k}) &= \left[\begin{pmatrix}\beta_1\\ m_{2k}\end{pmatrix}, \begin{pmatrix}\phi_1^2 & 0\\ 0 & s_{2k}^2\end{pmatrix}\right] \\ (\boldsymbol{\mu}_{21k}, \boldsymbol{\Sigma}_{21k}) &= \left[\begin{pmatrix}m_{1k}\\ \beta_2\end{pmatrix}, \begin{pmatrix}s_{1k}^2 & 0\\ 0 & \phi_2^2\end{pmatrix}\right] \\ (\boldsymbol{\mu}_{22k}, \boldsymbol{\Sigma}_{22k}) &= \left[\begin{pmatrix}m_{1k}\\ m_{2k}\end{pmatrix}, \begin{pmatrix}s_{1k}^2 & \kappa s_{1k}s_{2k}\\ \kappa s_{1k}s_{2k} & s_{2k}^2\end{pmatrix}\right] \end{aligned}$$

) $\boldsymbol{\pi}_t = (\omega_t, 1-\omega_t)$ and $\boldsymbol{\pi}_e = (\omega_e, 1-\omega_e)$ with prior exchangeability weights ω_t, ω_e on toxicity and efficacy respectively.

Now proof of the equivalence between this stipulation and the original one is as follows: First of all, we can similarly write the original E-BiEXNEX model for subtrial k as

$$\begin{aligned} \mathrm{Z} &\sim \mathcal{C}(\boldsymbol{\pi}_k) \\ \begin{pmatrix}\theta_k^t\\ \theta_k^e\end{pmatrix}\Bigg| \mathrm{Z}_k = l &\sim \mathcal{N}(\boldsymbol{\mu}_{lk}, \boldsymbol{\Sigma}_{lk}) \end{aligned} \tag{7}$$

where $l = 1,2,3,4$ denoting 4 components Section E-BiEXNEX model; $\boldsymbol{\mu}_{lk}$ and $\boldsymbol{\Sigma}_{lk}$ are corresponding mean and covariance matrices. Apparently $(\boldsymbol{\mu}_{1k}, \boldsymbol{\Sigma}_{1k}) = (\boldsymbol{\mu}_{11k}, \boldsymbol{\Sigma}_{11k})$, $(\boldsymbol{\mu}_{2k}, \boldsymbol{\Sigma}_{2k}) = (\boldsymbol{\mu}_{12k}, \boldsymbol{\Sigma}_{12k})$, $(\boldsymbol{\mu}_{3k}, \boldsymbol{\Sigma}_{3k}) = (\boldsymbol{\mu}_{21k}, \boldsymbol{\Sigma}_{21k})$, $(\boldsymbol{\mu}_{4k}, \boldsymbol{\Sigma}_{4k}) = (\boldsymbol{\mu}_{22k}, \boldsymbol{\Sigma}_{22k})$, and $\boldsymbol{\pi}_k = (\lambda_{1k}, \lambda_{2k}, \lambda_{3k}, \lambda_{4k})$.

In model 7, the posterior exchangeability weight for toxicity can be approximated by $\Pr\left(\mathrm{Z}_k = 1 \,\middle|\, \begin{pmatrix}\theta_k^t\\ \theta_k^e\end{pmatrix}\right) + \Pr\left(\mathrm{Z}_k = 2 \,\middle|\, \begin{pmatrix}\theta_k^t\\ \theta_k^e\end{pmatrix}\right)$ while in model 6 it is exactly $\Pr\left(\mathrm{Z}_{tk} = 1 \,\middle|\, \begin{pmatrix}\theta_k^t\\ \theta_k^e\end{pmatrix}\right)$. Next we show if $\Pr(Z_k = 1) = \Pr(Z_{tk} = 1, Z_{ek} = 1)$, $\Pr(Z_k = 2) = \Pr(Z_{tk} = 1, Z_{ek} = 2)$, $\Pr(Z_k = 3) = \Pr(Z_{tk} = 2, Z_{ek} = 1)$ and $\Pr(Z_k = 4) = \Pr(Z_{tk} = 2, Z_{ek} = 2)$ then

$$\Pr\left(\mathrm{Z}_{tk} = 1 \,\middle|\, \begin{pmatrix}\theta_k^t\\ \theta_k^e\end{pmatrix}\right) = \Pr\left(\mathrm{Z}_k = 1 \,\middle|\, \begin{pmatrix}\theta_k^t\\ \theta_k^e\end{pmatrix}\right) + \Pr\left(\mathrm{Z}_k = 2 \,\middle|\, \begin{pmatrix}\theta_k^t\\ \theta_k^e\end{pmatrix}\right) \tag{8}$$

Proof.
According to model 6,

$$\begin{aligned} \Pr\left(\mathrm{Z}_{tk} = 1 \,\middle|\, \begin{pmatrix}\theta_k^t\\ \theta_k^e\end{pmatrix}\right) &= \sum_{r=1}^{2} \Pr\left(\mathrm{Z}_{tk} = 1, \mathrm{Z}_{ek} = r \,\middle|\, \begin{pmatrix}\theta_k^t\\ \theta_k^e\end{pmatrix}\right) \\ &= \frac{\sum_{r=1}^{2} \Pr(\mathrm{Z}_{tk} = 1, \mathrm{Z}_{ek} = r) \Pr\left(\begin{pmatrix}\theta_k^t\\ \theta_k^e\end{pmatrix}\middle| \mathrm{Z}_{tk} = 1, \mathrm{Z}_{ek} = r\right)}{\sum_{i,j=1}^{2} \Pr(\mathrm{Z}_{tk} = i, \mathrm{Z}_{ek} = j) \Pr\left(\begin{pmatrix}\theta_k^t\\ \theta_k^e\end{pmatrix}\middle| \mathrm{Z}_{tk} = i, \mathrm{Z}_{ek} = j\right)} \end{aligned}$$

while model 7 shows

$$\sum_{r=1}^{2} \Pr\left(\mathrm{Z}_k = r \,\middle|\, \begin{pmatrix}\theta_k^t\\ \theta_k^e\end{pmatrix}\right) = \frac{\sum_{r=1}^{2} \Pr(\mathrm{Z}_k = r) \Pr\left(\begin{pmatrix}\theta_k^t\\ \theta_k^e\end{pmatrix}\middle| \mathrm{Z}_k = r\right)}{\sum_{l=1}^{4} \Pr(\mathrm{Z}_k = l) \Pr\left(\begin{pmatrix}\theta_k^t\\ \theta_k^e\end{pmatrix}\middle| \mathrm{Z}_k = l\right)}$$

Due to the relation of likelihoods between the two models, it is obvious that if the previous equations for prior probabilities of Z_k and (Z_{tk}, Z_{ek}) hold, the two sides in equation 8 will be strictly equal, indicating that the two models are identical under very simple constraints concerning prior exchangeability weights.

Single-arm models

Single-arm data models

We consider a single-arm basket trial with K subtrials. Only the experimental group ($j = E$) is observed. We keep the same symbols as in the randomised setting; the only change is that the data likelihood is written for the observed arm $j = E$ only.

Toxicity (binary) For subtrial $k = 1, \ldots, K$, let y_{Ek} be the number of toxicity events among n_{Ek} patients, with probability p_{Ek}. The single-arm toxicity likelihood is

$$\begin{aligned} y_{Ek} &\sim \mathrm{Binomial}(n_{Ek}, p_{Ek}), \\ \mathrm{logit}(p_{Ek}) &= \alpha_k^t + \theta_k^t. \end{aligned} \tag{9}$$

Compared with the randomised model $\mathrm{logit}(p_{jk}) = \alpha_k^t + T(j)\,\theta_k^t$, this is obtained by restricting to the observed arm $j = E$ (so that $T(E) = 1$). In the single-arm setting, α_k^t represents a reference baseline log-odds for subtrial k.

Efficacy (continuous) Let z_{iEk} be the continuous efficacy outcome for patient i in subtrial k. The single-arm efficacy likelihood is

$$\begin{aligned} z_{iEk} &\sim \mathcal{N}(\mu_{Ek}, \sigma_{Ek}^2), \\ \mu_{Ek} &= \alpha_k^e + \theta_k^e. \end{aligned} \tag{10}$$

Compared with the randomised model $\mu_{jk} = \alpha_k^e + T(j)\,\theta_k^e$, this is again obtained by restricting to $j = E$ (so that $T(E) = 1$). In the single-arm setting, α_k^e represents a reference baseline mean for subtrial k.

Single-arm BiEXNEX model

The BiEXNEX prior model for the subtrial-specific parameter vector $(\theta_k^t, \theta_k^e)^\top$ is unchanged; only the likelihood in Section 1 differs in the single-arm setting. For each subtrial k:
with prior probability ω_k, parameter vectors are exchangeable (EX):

$$\begin{pmatrix} \theta_k^t \\ \theta_k^e \end{pmatrix} \Bigg| \begin{pmatrix} \beta_1 \\ \beta_2 \end{pmatrix}, \begin{pmatrix} \phi_1 \\ \phi_2 \end{pmatrix}, \rho \sim \mathcal{N}\left(\begin{pmatrix} \beta_1 \\ \beta_2 \end{pmatrix}, \begin{pmatrix} \phi_1^2 & \rho\phi_1\phi_2 \\ \rho\phi_1\phi_2 & \phi_2^2 \end{pmatrix} \right), \tag{11a}$$

with prior probability $1 - \omega_k$, parameter vectors are non-exchangeable (NEX):

$$\begin{pmatrix} \theta_k^t \\ \theta_k^e \end{pmatrix} \Bigg| \kappa \sim \mathcal{N}\left(\begin{pmatrix} m_{1k} \\ m_{2k} \end{pmatrix}, \begin{pmatrix} s_{1k}^2 & \kappa s_{1k}s_{2k} \\ \kappa s_{1k}s_{2k} & s_{2k}^2 \end{pmatrix} \right). \tag{11b}$$

Single-arm E-BiEXNEX model

The E-BiEXNEX prior model for $(\theta_k^t, \theta_k^e)^\top$ is also unchanged; only the likelihood in Section 1 differs in the single-arm setting. For each subtrial k, the four-component mixture prior is:
with probability λ_{1k}, both θ_k^t and θ_k^e are exchangeable across subtrials,

$$\begin{pmatrix} \theta_k^t \\ \theta_k^e \end{pmatrix} \Bigg| \begin{pmatrix} \beta_1 \\ \beta_2 \end{pmatrix}, \begin{pmatrix} \phi_1 \\ \phi_2 \end{pmatrix}, \rho \sim \mathcal{N}\left(\begin{pmatrix} \beta_1 \\ \beta_2 \end{pmatrix}, \begin{pmatrix} \phi_1^2 & \rho\phi_1\phi_2 \\ \rho\phi_1\phi_2 & \phi_2^2 \end{pmatrix} \right), \tag{12a}$$

with probability λ_{2k}, only θ_k^t is exchangeable,

$$\begin{pmatrix} \theta_k^t \\ \theta_k^e \end{pmatrix} \Bigg| \beta_1, \phi_1 \sim \mathcal{N}\left(\begin{pmatrix} \beta_1 \\ m_{2k} \end{pmatrix}, \begin{pmatrix} \phi_1^2 & 0 \\ 0 & s_{2k}^2 \end{pmatrix} \right), \tag{12b}$$

with probability λ_{3k}, only θ_k^e is exchangeable,

$$\begin{pmatrix} \theta_k^t \\ \theta_k^e \end{pmatrix} \Bigg| \beta_2, \phi_2 \sim \mathcal{N}\left(\begin{pmatrix} m_{1k} \\ \beta_2 \end{pmatrix}, \begin{pmatrix} s_{1k}^2 & 0 \\ 0 & \phi_2^2 \end{pmatrix} \right), \tag{12c}$$

with probability λ_{4k}, neither θ_k^t nor θ_k^e is exchangeable,

$$\begin{pmatrix} \theta_k^t \\ \theta_k^e \end{pmatrix} \Bigg| \kappa \sim \mathcal{N}\left(\begin{pmatrix} m_{1k} \\ m_{2k} \end{pmatrix}, \begin{pmatrix} s_{1k}^2 & \kappa s_{1k}s_{2k} \\ \kappa s_{1k}s_{2k} & s_{2k}^2 \end{pmatrix} \right), \tag{12d}$$

where $\sum_{i=1}^4 \lambda_{ik} = 1$ and $0 \le \lambda_{ik} \le 1$ for each subtrial k.

The response rates for control and treatment groups under each scenario for power calculation

Table 3. p_{Ek} specification table with 6 scenarios and $K = 6$ subtrials. The bottom row indicates p_{Ck} for all subtrials.

Scenario	subtrial k with (half-)sample size $n_{Ck} = n_{Ek}$, e.g. 1 (10)					
	1 (10)	2 (10)	3 (10)	4 (6)	5 (12)	6 (10)
Ia	0.19	0.14	0.26	0.29	0.20	0.24
Ib	0.17	0.12	0.19	0.52	0.11	0.09
IIa	0.11	0.56	0.63	0.09	0.43	0.26
IIb	0.11	0.56	0.23	0.09	0.44	0.63
IIIa	0.10	0.77	0.63	0.34	0.03	0.98
IIIb	0.14	0.09	0.17	0.21	0.17	0.19
p_{Ck}	0.34	0.24	0.41	0.46	0.40	0.43

Table 4. μ_{Ek} specification table with 6 scenarios and $K = 6$ subtrials, with the bottom row showing μ_{Ck} for all subtrials.

Scenario	subtrial k with (half-)sample size $n_{Ck} = n_{Ek}$, e.g. 1 (10)					
	1 (10)	2 (10)	3 (10)	4 (6)	5 (12)	6 (10)
Ia	3.96	4.32	3.71	3.60	3.80	3.62
Ib	3.97	4.23	3.82	4.68	2.59	1.76
IIa	1.43	3.55	3.70	4.68	2.59	5.08
IIb	4.63	1.84	4.10	4.34	3.03	1.12
IIIa	4.20	4.50	4.02	3.88	4.05	3.96
IIIb	1.93	6.98	5.80	2.86	4.20	2.84
μ_{Ck}	3.20	3.50	3.02	2.88	3.05	2.96

Overall error rates

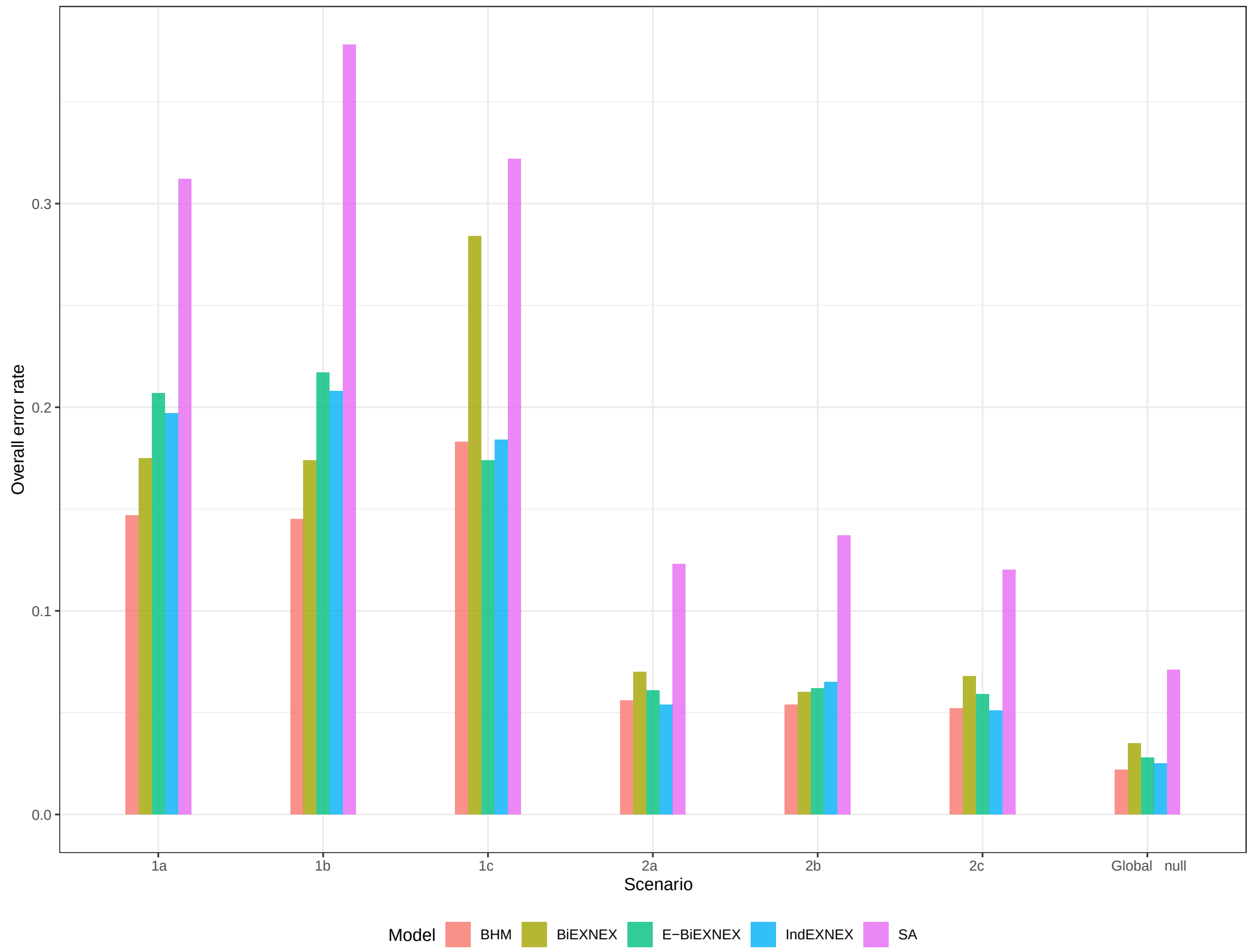

Figure 6. The overall error rate under each null scenario for the first decision rule.

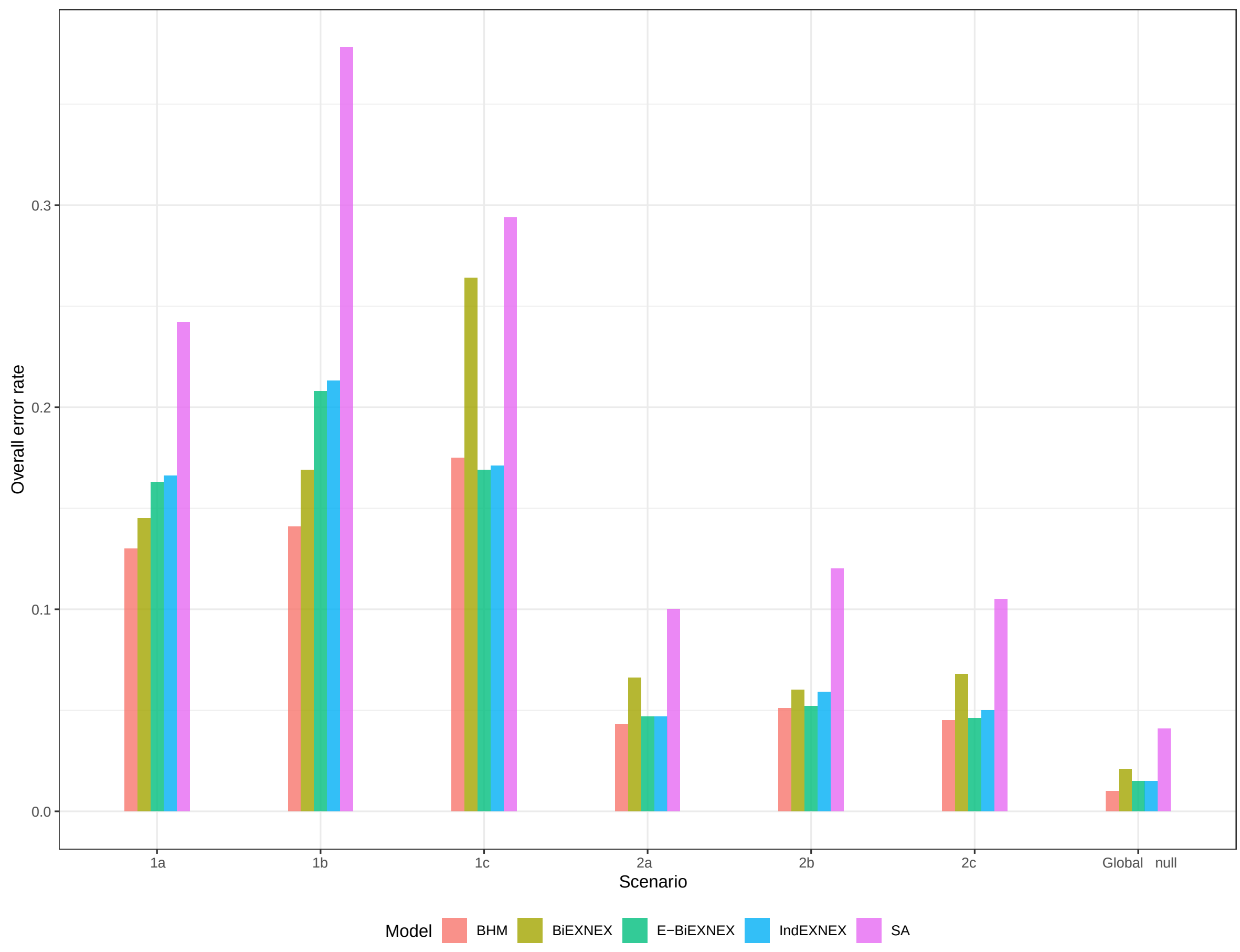

Figure 7. The overall error rate under each null scenario for the second decision rule.

Sensitivity analysis with smaller sample sizes

In this section, our objective is to conduct a sensitive analysis to test how changing sample sizes can influence power calculation in presence of asynchronous exchangeability of treatment effects. We add more varieties as new sub-scenarios of original power scenario IIIa and IIIb with different degrees of similarity:

- Sub-scenarios based on IIIa ($\theta_k^e = 1.0$):
  - 1.1: $\boldsymbol{\theta}^t = (-0.69, -1.2, -1.24, -0.74, -1.04, -0.8)$ with $\text{SD}(\boldsymbol{\theta}^t) \approx 0.25$
  - 1.2: $\boldsymbol{\theta}^t = (-0.97, -0.6, -1.09, -0.48, 0.38, -1.72)$ with $\text{SD}(\boldsymbol{\theta}^t) \approx 0.75$
  - 1.3: $\boldsymbol{\theta}^t = (-3.82, -1.24, -0.4, 0.12, 0.11, 0.36)$ with $\text{SD}(\boldsymbol{\theta}^t) \approx 1.50$
  - 1.3: $\boldsymbol{\theta}^t = (-2.46, 4.17, -0.28, -2.64, 0.79, -2.83)$ with $\text{SD}(\boldsymbol{\theta}^t) \approx 3.00$
- Sub-scenarios based on IIIb ($\theta_k^t = -1.2$):
  - 2.1: $\boldsymbol{\theta}^e = (0.77, 1.40, 1.15, 0.86, 1.25, 0.90)$ with $\text{SD}(\boldsymbol{\theta}^e) \approx 0.25$
  - 2.2: $\boldsymbol{\theta}^e = (2.15, 0.36, 1.08, 1.86, 0.89, 0.47)$ with $\text{SD}(\boldsymbol{\theta}^e) \approx 0.75$
  - 2.3: $\boldsymbol{\theta}^e = (2.86, 1.27, -1.51, -0.59, -0.68, 0.43)$ with $\text{SD}(\boldsymbol{\theta}^e) \approx 1.50$
  - 2.4: $\boldsymbol{\theta}^e = (-1.26, 4.70, -3.55, 0.09, -3.18, 2.13)$ with $\text{SD}(\boldsymbol{\theta}^e) \approx 3.00$

Each series of sub-scenarios under IIIa (or IIIb) have large to small similarities where the shared information in θ_k^t (or θ_k^e) gradually diminishes. The (half-)sample sizes for subtrials are (5, 5, 5, 3, 6, 5), and all other parameters, priors and decision rules used for each method are identical as in Section Simulation Study to produce comparable results.

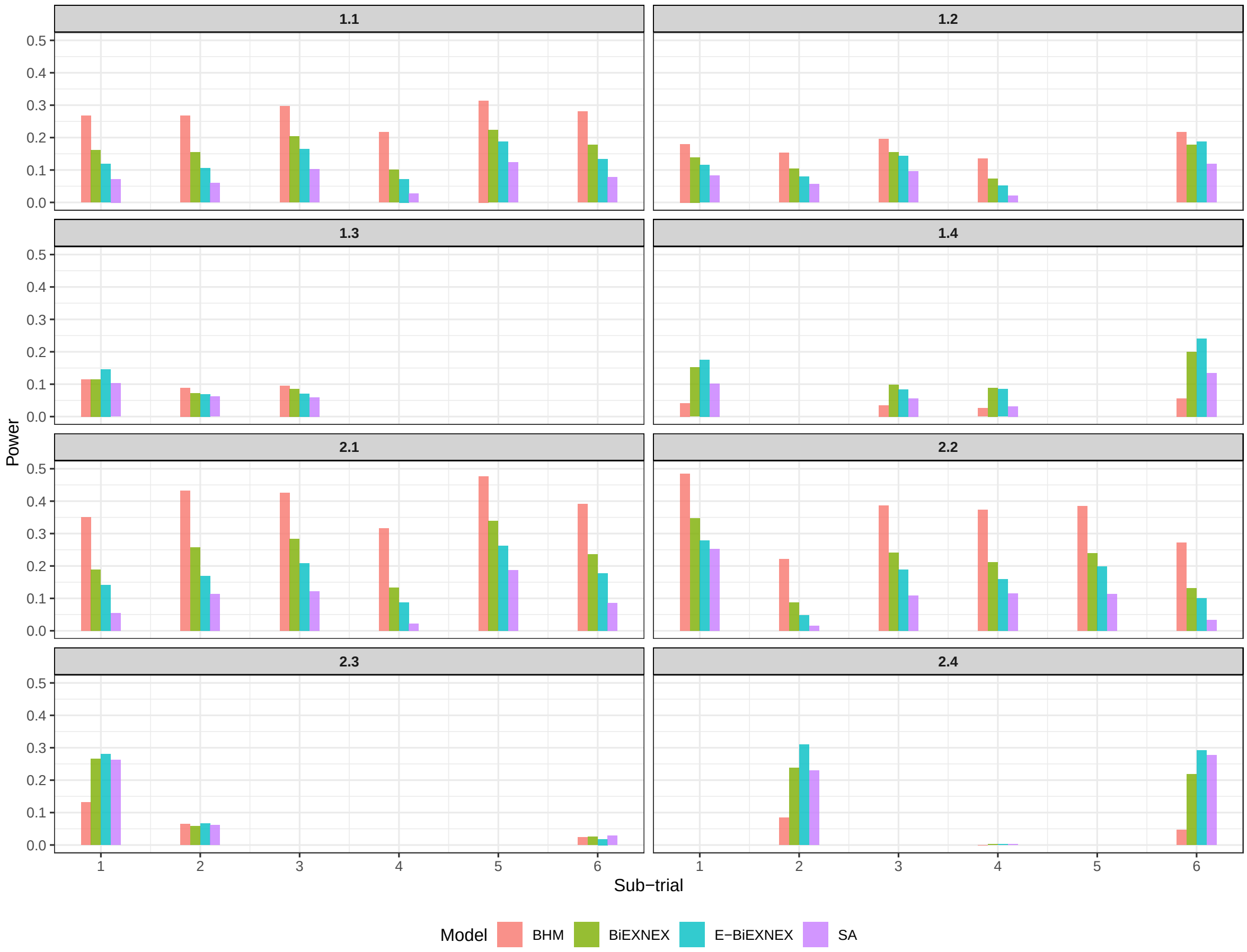

Figure 8. Bayesian analogues of power for the smaller sample sizes.

Figure 8 shows that regardless of which treatment effect is the same across all subtrials, BHM will achieve lower power when the similarity of another treatment effect decreases while E-BiEXNEX can gradually strike the highest power among all methods (e.g. from 1.1 to 1.4 or 2.1 to 2.4). In contrast to the scenario IIIb in Section Results where θ_k^t are the same for all subtrials and θ_k^e are largely different, sub-scenarios 2.3 and 2.4 in this section present a reverse pattern, proving that the smaller sample sizes cannot provide sufficient information even for continuous outcomes. Hence, borrowing of information depends on both treatment effects.

Large-sample sensitivity analysis

We retained the six power scenarios in Table 2, the same prior specifications, and the same joint Go/No-Go decision rule as in the main simulation study. The only change was to increase the sample size to $n_{Ck} = n_{Ek} = 50$ for each subtrial. All five methods, BHM, BiEXNEX, E-BiEXNEX, IndEXNEX and SA, were re-fitted under the same settings.

Figure 9 shows the Go decision rates under the large-sample setting. As in Figure 5 of the main manuscript, bars are shown only for subtrials where the treatment is truly favourable, defined by $\theta_k^t < 0$ and $\theta_k^e > \delta$. Using larger sample sizes can clearly improve power across most true-positive subtrials. For example, in scenario Ia, where both toxicity and efficacy effects are similar across subtrials, E-BiEXNEX achieved Go decision rates of 0.741, 0.607, 0.706, 0.753, 0.858 and 0.826 across the six subtrials. In scenario IIIb, where toxicity effects are exchangeable but efficacy effects vary, E-BiEXNEX achieved Go decision rates of 0.878, 0.944 and 0.937 in the true-positive subtrials.

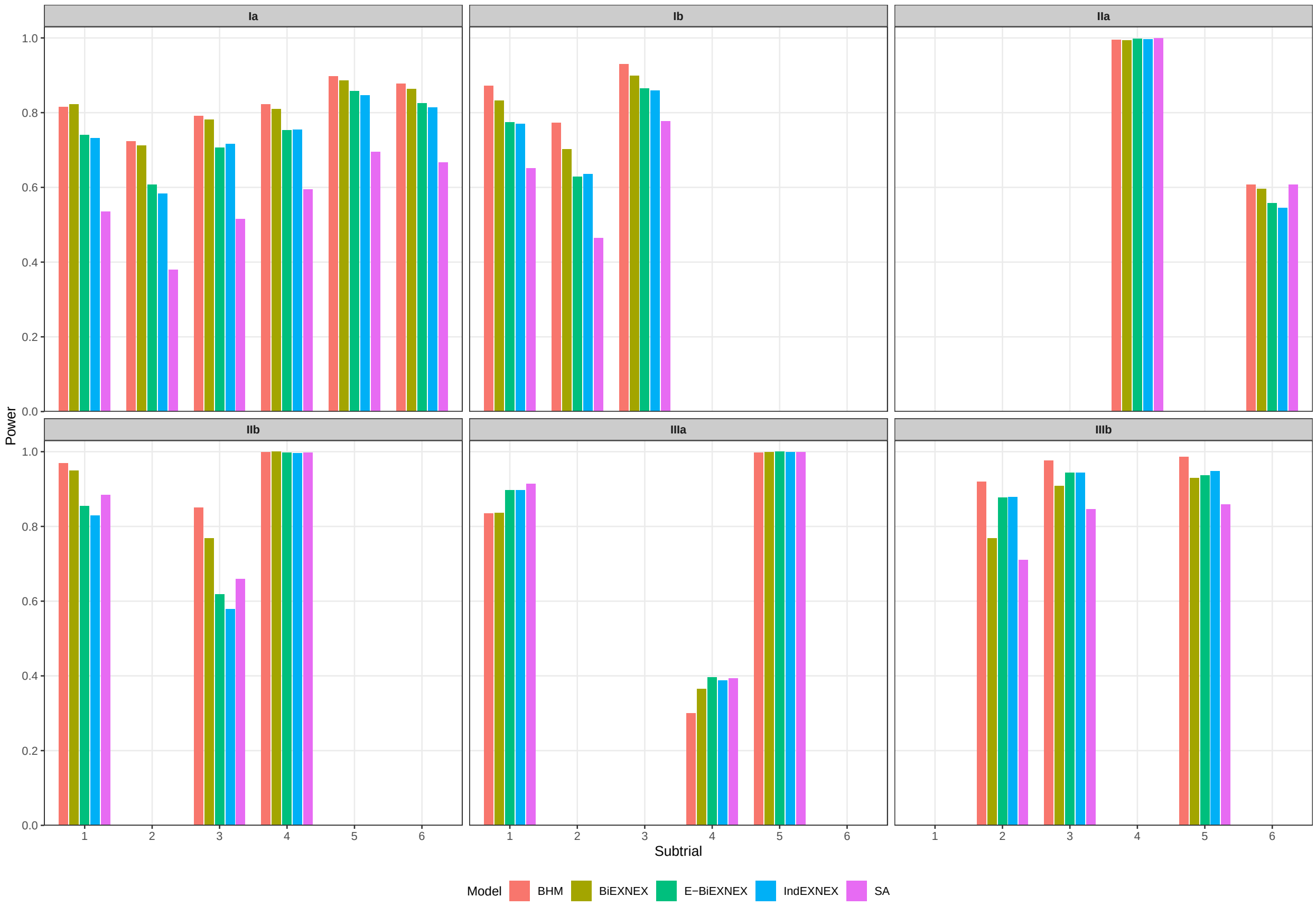

Figure 9. Large-sample sensitivity analysis. Bayesian analogues of power are shown only for true-positive subtrials, defined by $\theta_k^t < 0$ and $\theta_k^e > \delta$, matching the convention used in Figure 5 in the main manuscript.

However, not all true-positive subtrials reached high power. This occurred mainly in naturally difficult or borderline cases where one treatment effect was close to the joint decision boundary. These results indicate that larger sample sizes can definitely improve the absolute power, but that achieving conventional power levels remains scenario-dependent and clinical specific under a joint toxicity–efficacy decision rule. Therefore, in practical applications, sample size and decision thresholds should be carefully calibrated.

Simulation results under the first decision rule

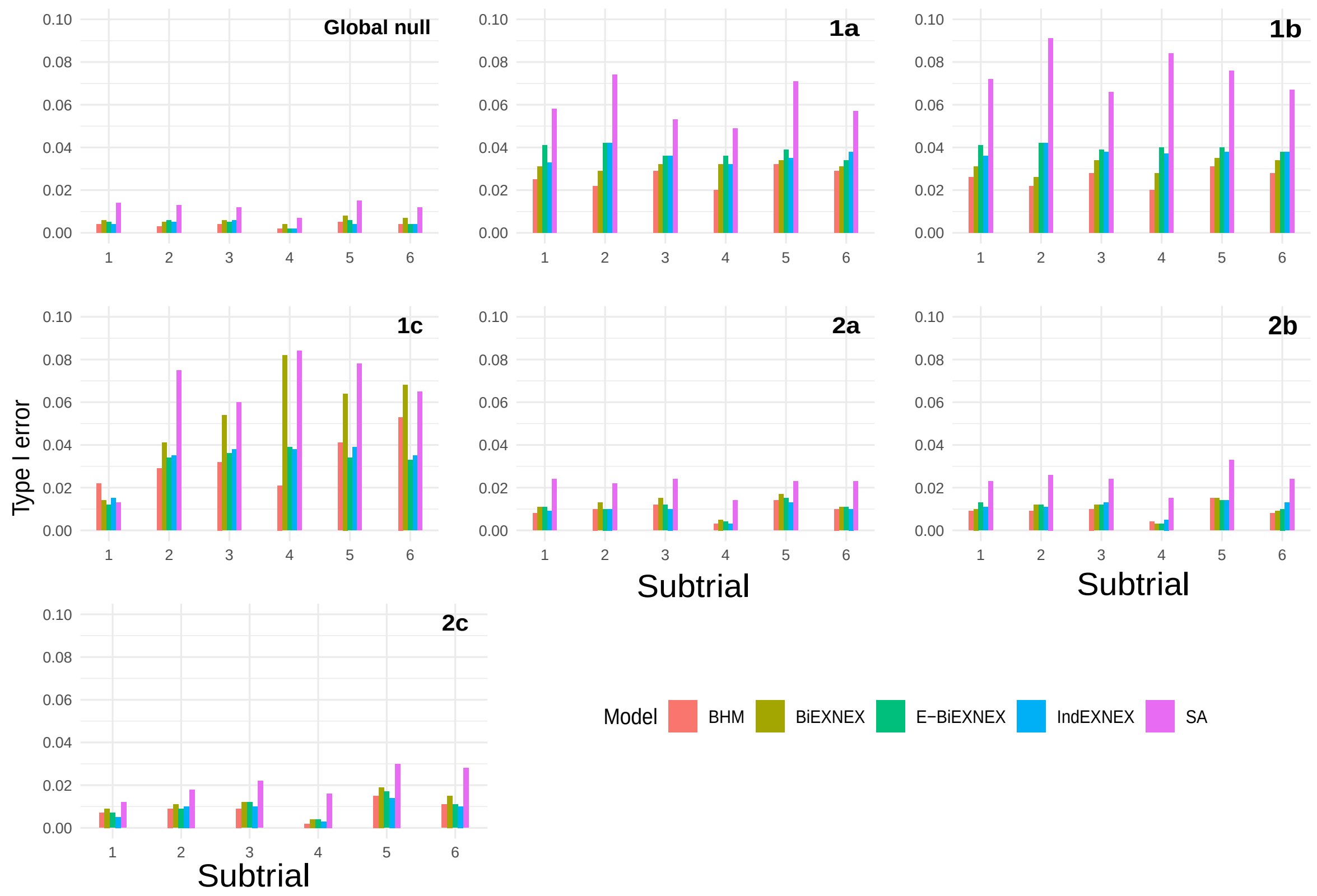

Figure 10. Bayesian analogues of type I error rates for each null scenario.

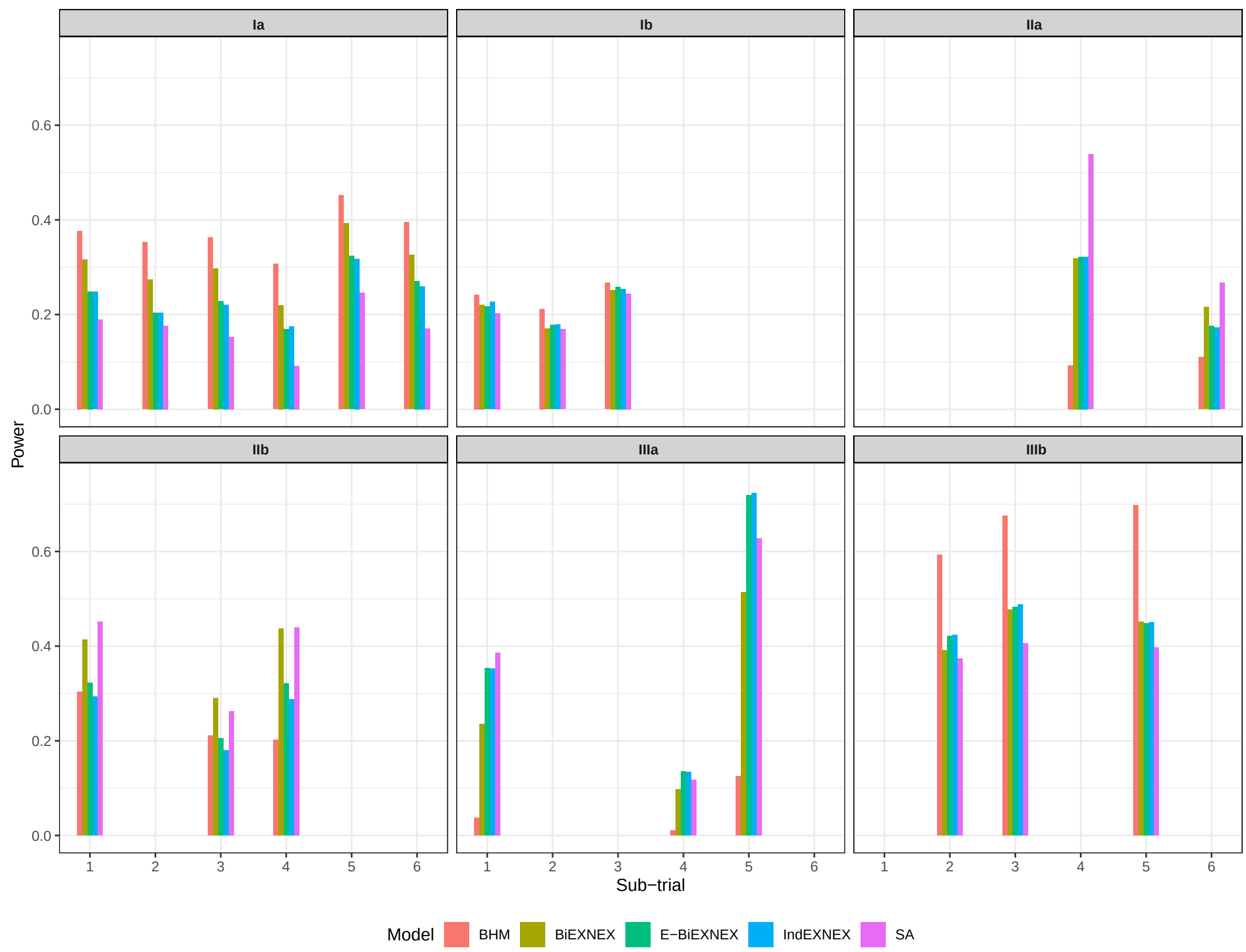

Figure 11. Bayesian analogues of power for each scenario, model, and subtrial.

Sensitivity analysis: fully correlated structure in E-BiEXNEX

We conducted a sensitivity analysis to compare how correlation structure of E-BiEXNEX will impact the probability of Go decisions. We abbreviate the proposed E-BiEXNEX (see Section Methods) to pEB, and the fully correlated E-BiEXNEX (see the following model) to fEB. The fEB is established by directly imposing unknown correlation parameters to component (4b) and (4c) in pEB:

$$\begin{pmatrix}\theta_k^t\\ \theta_k^e\end{pmatrix}\Bigg|, \begin{pmatrix}\beta_1\\ \beta_2\end{pmatrix}, \begin{pmatrix}\phi_1\\ \phi_2\end{pmatrix}, \rho \sim \mathcal{N}\left(\begin{pmatrix}\beta_1\\ \beta_2\end{pmatrix}, \begin{pmatrix}\phi_1^2 & \rho\phi_1\phi_2\\ \rho\phi_1\phi_2 & \phi_2^2\end{pmatrix}\right) \tag{13a}$$

with probability λ_{2k}, only θ_k^t is exchangeable,

$$\begin{pmatrix}\theta_k^t\\ \theta_k^e\end{pmatrix}\Bigg| \beta_1, \phi_1 \sim \mathcal{N}\left(\begin{pmatrix}\beta_1\\ m_{2k}\end{pmatrix}, \begin{pmatrix}\phi_1^2 & \gamma_1\phi_1 s_{2k}\\ \gamma_1\phi_1 s_{2k} & s_{2k}^2\end{pmatrix}\right) \tag{13b}$$

with probability λ_{3k}, only θ_k^e is exchangeable,

$$\begin{pmatrix}\theta_k^t\\ \theta_k^e\end{pmatrix}\Bigg| \beta_2, \phi_2 \sim \mathcal{N}\left(\begin{pmatrix}m_{1k}\\ \beta_2\end{pmatrix}, \begin{pmatrix}s_{1k}^2 & \gamma_2 s_{1k}\phi_2\\ \gamma_2 s_{1k}\phi_2 & \phi_2^2\end{pmatrix}\right) \tag{13c}$$

with probability λ_{4k}, neither θ_k^t nor θ_k^e is exchangeable,

$$\begin{pmatrix}\theta_k^t\\ \theta_k^e\end{pmatrix}\Bigg| \kappa \quad\sim\quad \mathcal{N}\left(\begin{pmatrix}m_{1k}\\ m_{2k}\end{pmatrix}, \begin{pmatrix}s_{1k}^2 & \kappa s_{1k}s_{2k}\\ \kappa s_{1k}s_{2k} & s_{2k}^2\end{pmatrix}\right) \tag{13d}$$

We then give a uniform prior $\mathcal{U}(-1, 1)$ to all correlation parameters in pEB and fEB. We chose the plausibly fEB-advantageous scenarios (Table 5) where θ_k and θ_k^t are highly correlated and only one can be exchangeable while the other is not, because pEB does not consideration the correlation in these scenarios.

Table 5. Scenario setting used for testing whether the incorporation of fully correlated structure in E-BiEXNEX leads to improvement of Go decision rate.

Scenario		Subtrial k						Description
		1 (10)	2 (10)	3 (10)	4 (6)	5 (12)	6 (10)	
S1	θ_k^t	-1.5	-1.45	-1.40	-1.35	-1.30	-1.25	Similar θ_k^t and varied θ_k^e. Cor$(\theta_k^t, \theta_k^e) \approx 0.99$
	θ_k^e	-2.15	-1.04	0.02	1.10	1.84	2.97	
S2	θ_k^t	-2.96	-2.46	-1.10	0.17	0.44	1.78	Similar θ_k^e and varied θ_k^t. Cor$(\theta_k^t, \theta_k^e) \approx 0.98$
	θ_k^e	0.55	0.60	0.65	0.70	0.75	0.80	

Table 6 shows that, under 5000 simulations, there is no obvious difference in in terms of Go decision rate between pEB and fEB in scenarios S1 and S2, not to mention other scenarios which can be explained by both pEB and fEB. Therefore, we think using pEB is sufficient to fit patient outcomes.

Table 6. Go decision rate of scenario S1 and S2 for pEB and fEB given 5000 simulations.

Scenario	Model	Go decision rate of subtrial k					
		1 (10)	2 (10)	3 (10)	4 (6)	5 (12)	6 (10)
S1	pEB	0	0	0.039	0.164	0.559	0.530
	fEB	0	0	0.034	0.168	0.559	0.529
S2	pEB	0.260	0.175	0.174	0.033	0.020	0.001
	fEB	0.257	0.177	0.172	0.027	0.019	0.001

Sensitivity analysis: SA with and without between-subtrial correlation

To assess whether SA's type I error inflation in all null scenarios could be driven by the working-independence assumption, we compared (i) the proposed SA (pSA) specification used in the main paper (no borrowing and zero correlation between (θ_k^t, θ_k^e)) and (ii) a variant that retains between-subtrial parameter correlation between (θ_k^t, θ_k^e) (correlated SA, hereafter abbreviated to cSA), i.e. equal to only setting $\omega_k = 0$ for all k in BiEXNEX. Table 7 shows that, under 5000 simulations, allowing correlation did not reduce type I error generally, indicating that the main conclusions are not sensitive to this aspect of the SA comparator.

Table 7. The comparison of type I error rate of all null scenarios between pSA and cSA

Scenario	Model	Type I error of subtrial k 1 (10)	2 (10)	3 (10)	4 (6)	5 (12)	6 (10)
Global Null	pSA	0.008	0.008	0.009	0.006	0.006	0.008
	cSA	0.014	0.008	0.009	0.010	0.013	0.010
1a	pSA	0.043	0.048	0.042	0.035	0.042	0.041
	cSA	0.048	0.056	0.054	0.042	0.063	0.052
1b	pSA	0.079	0.094	0.070	0.083	0.077	0.059
	cSA	0.072	0.082	0.072	0.083	0.069	0.071
1c	pSA	0.009	0.046	0.058	0.078	0.072	0.062
	cSA	0.009	0.046	0.061	0.082	0.069	0.066
2a	pSA	0.020	0.017	0.018	0.012	0.022	0.018
	cSA	0.018	0.012	0.022	0.015	0.024	0.023
2b	pSA	0.020	0.020	0.024	0.016	0.032	0.025
	cSA	0.021	0.018	0.027	0.015	0.032	0.022
2c	pSA	0.008	0.010	0.021	0.010	0.027	0.023
	cSA	0.007	0.013	0.019	0.012	0.031	0.026

Code availability

For readers who are interested in code used for data-generation, model fitting, and the simulation study, we have uploaded relevant code to an open source repository: E-BiEXNEX related R&JAGS code.
